# Adaptive Sampling of Information in Perceptual Decision-Making

**DOI:** 10.1371/journal.pone.0078993

**Published:** 2013-11-27

**Authors:** Thomas C. Cassey, David R. Evens, Rafal Bogacz, James A. R. Marshall, Casimir J. H. Ludwig

**Affiliations:** 1 Department of Computer Science, University of Bristol, Bristol, United Kingdom; 2 School of Experimental Psychology, University of Bristol, Bristol, United Kingdom; 3 Department of Computer Science and Kroto Research Institute, University of Sheffield, Sheffield, United Kingdom; University of Sussex, United Kingdom

## Abstract

In many perceptual and cognitive decision-making problems, humans sample multiple noisy information sources serially, and integrate the sampled information to make an overall decision. We derive the optimal decision procedure for two-alternative choice tasks in which the different options are sampled one at a time, sources vary in the quality of the information they provide, and the available time is fixed. To maximize accuracy, the optimal observer allocates time to sampling different information sources in proportion to their noise levels. We tested human observers in a corresponding perceptual decision-making task. Observers compared the direction of two random dot motion patterns that were triggered only when fixated. Observers allocated more time to the noisier pattern, in a manner that correlated with their sensory uncertainty about the direction of the patterns. There were several differences between the optimal observer predictions and human behaviour. These differences point to a number of other factors, beyond the quality of the currently available sources of information, that influences the sampling strategy.

## Introduction

In humans and many other biological organisms, the sensory and cognitive machinery used to pick up and process information from the environment is extremely limited. For the human visual system, high resolution vision is only possible in the small part of the visual scene that projects onto the fovea. As a result, we sample information selectively and sequentially from the visual world by frequently shifting the line of sight [1]. We are often presented with perceptual decision problems which involve sampling information from multiple sources and then using that information to make an overall judgement about the “state of the world”. For example, when crossing a road, we must integrate information from either direction, sampled in serial, to decide whether it is safe to cross.

Sensory information is typically noisy, and therefore uncertain [2], [3]. The level of uncertainty may vary across different information sources (e.g., the view of the road in one direction may be obstructed by a tree). In different situations (e.g., familiar versus unfamiliar junctions) we may have different amounts of prior knowledge of such variations in information quality. Internal processing mechanisms in the brain will insert additional noise [4], [5]. Switching between information sources incurs a temporal cost, in the form of reduced visual sensitivity for a period during and around the movement from one source to another [6]–[8]. A central challenge in such decision problems is to allocate a limited amount of time appropriately to different sources of information that can only be sampled one at a time.

**Figure 1:**
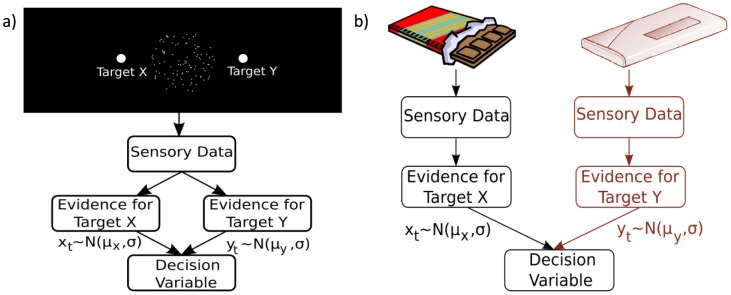
Models of the decision process for two different two-alternative decision problems. (a) Decision model for a single source two-alternative forced choice decision. A frequently used stimulus in such tasks is the random dot kinematogram (RDK), which consists of a number of dots, only some of which move in a particular “signal” direction. Subjects are typically asked to decide in which of two directions the RDK is moving. With a single source of visual information the sensory data provide evidence for both alternatives. In motion discrimination tasks this evidence comes from neurons in area MT whose activity (firing rate) is tuned to respond to motion in a particular direction. At each moment in time, evidence from two populations of neurons is used to update a single decision variable. (b) Decision model proposed by Krajbich et al. [18] for a comparative two-alternative decision problem with two sources of visual stimuli. In their study, participants were asked to choose between two food items presented simultaneously in different locations on the screen. With each stimulus providing evidence about one particular alternative, the decision variable is no longer updated simultaneously by evidence for all available targets. Instead, as a target is fixated (target X in (b)) evidence supporting that target is generated and incorporated into the decision variable. During this time, the mechanisms that represent the evidence for the non-fixated target remain silent (shaded out branch for target Y in (b)).

Much of the work on perceptual decision-making involves observers (humans or non-human primates) making a binary decision about a single stimulus [9], [10]. For instance, observers may view a noisy pattern and make a decision about the average direction of motion of the pattern (typically either to the left or right). In such tasks it has been observed that certain neurons in frontal and parietal areas integrate information from sensory areas that encode the evidence for alternative choices [11], [12]. It has been proposed [13]–[16] that these neural circuits implement the computation described by the diffusion model (Figure 1a) [17]. This model assumes that the brain computes a decision variable that corresponds to the integrated difference between inputs from sensory neurons selective for the two alternatives. The diffusion model has been shown, under certain assumptions, to be the continuum limit of sequentially computing the ratio of the likelihoods of the two alternatives [18], which can be used to form optimal decisions in fixed time and free-response decision problems [19], [20].

Recently, a modification of the diffusion model has been proposed which mechanistically describes choice process between two options that are inspected serially [21] (Figure 1b). In this modified diffusion model, the average rate of change of the decision variable depends on which option is currently sampled (i.e. fixated). In line with the predictions of the model, it was observed that the cumulative length of visual fixations on each target influenced the outcome of the decision process, with choices biased towards the option that had been viewed for longer.

In this paper, we consider a comparative decision problem in which multiple sources of information have to be sampled sequentially, in a fixed period of time, to make a two-alternative forced choice decision. Critically, we consider the situation in which different sources of information have different levels of noise. Our specific aims are threefold. First, given the novelty of the decision problem under investigation, we identified the optimal sampling strategy that maximises accuracy in this type of situation. Second, we developed an experimental paradigm that extends the classic perceptual decision-making task described above to this more challenging situation. We assessed how human sampling behaviour compares to the qualitative predictions generated by the ideal observer. Third, we examined the relation between the normative model developed below and the fixation-dependent drift diffusion model that has been applied to the specific instance of comparative choice described above [21].

## Results

### Optimal observer

As with previous two-alternative perceptual decision studies [9], [10], we assume that evidence for each alternative is encoded in the firing rates of neurons responding to the visual stimuli. As in previous studies [13], [14], for simplicity we assume that the average firing rate of a population of neurons selective for a particular stimulus has a normal distribution across different time intervals during which a given stimulus is presented. This assumption can be made because the firing rate of a neural population within an interval is an average of the rates of many individual neurons and, according to the central limit theorem, can be approximated by a normal distribution. We assume these firing rates are integrated until the available time has elapsed and the decision is determined by the state of the integrated decision variable (Figure 1). For the ideal observer we treat the firing rate of the neurons responding to a given visual stimulus as an independent normally distributed random variable, which we denote by 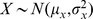 and 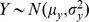 for each of the two information sources. The mean firing rates, 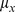 and 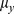, are assumed to be linked to the strength of evidence for each alternative. Finally, we denote the samples drawn from 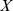 and 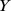 by 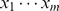 and 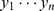 respectively, where *m* and *n* denote the numbers of samples obtained from *X* and *Y*.

We assume that one source of information will evoke a greater response in terms of average firing rate than the other. A comparative decision can be formulated as a decision between two hypotheses, 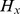 and 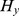 which are shown below:
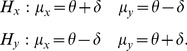
(1)

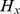
 is the hypothesis that the sampled evidence supporting alternative 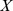 outweighs that supporting alternative 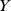 and 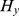 is the hypothesis that the sampled evidence supporting alternative 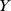 outweighs that supporting alternative 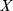. We assume that the observer has learned the relative difference between the two sources, which we denote by: 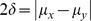. In the context of the experimental work reported below, it seems reasonable that observers would acquire this knowledge after experiencing a number of trials. Importantly, we assume that the observer does *not* know the average of the means of the two sources which we denote by *θ*. Thus, Equation 1 embodies a comparative decision rule: the response evoked by one alternative cannot be used to choose between these two hypotheses (i.e., just “knowing” the mean value of one source is not sufficient).

Deciding which hypothesis to select on the basis of the available evidence can be performed optimally using a log-likelihood ratio test which compares the value of the log-likelihood ratio, denoted 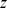
_,_ to some problem dependent decision criterion 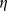, with 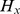 chosen when above the criterion and 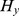 chosen otherwise. In the case where both hypotheses are equally likely with 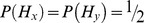 the optimal decision criterion is 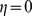 and the sign of 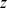 determines the hypothesis to select with 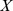 chosen when 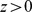 and 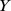 chosen when 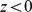. The log-likelihood ratio for the above problem was derived by Hayre and Gittins [22] (see Materials S1 for full derivation). The derivation is long and complicated because Hayre and Gittins avoided making any assumption about the unknown parameter *θ*. However, the same value of the log-likelihood ratio can be easily obtained in a special case where one assumes that all values of *θ* are equally likely. We outline this simplified derivation below. Assuming that *θ* has a uniform distribution on the interval between −*B* and *B*, the likelihood of observed samples given hypothesis *H_x_* is equal to:
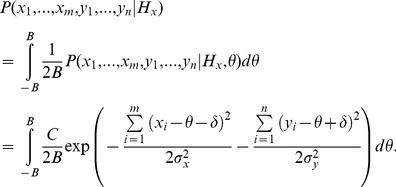
(2)where 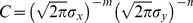. Equation 2 can be rearranged to factor out *θ*:
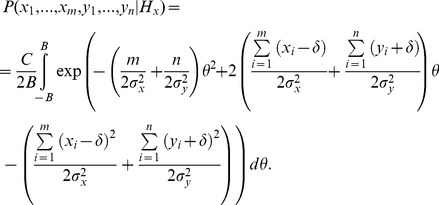
(3)Assuming that *B* is large, the above equation can be expressed without the unknown parameter *θ* using the following identity:
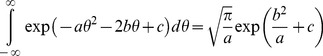
(4)Expressing the likelihood of samples given *H_y_* in an analogous way and taking the ratio of the likelihoods, most of the terms cancel and the log likelihood ratio becomes:
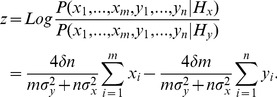
(5)According to Equation 5, at each interval in the decision process, the log-likelihood ratio is dependent on the difference in the weighted summation of evidence from each alternative.

**Figure 2:**
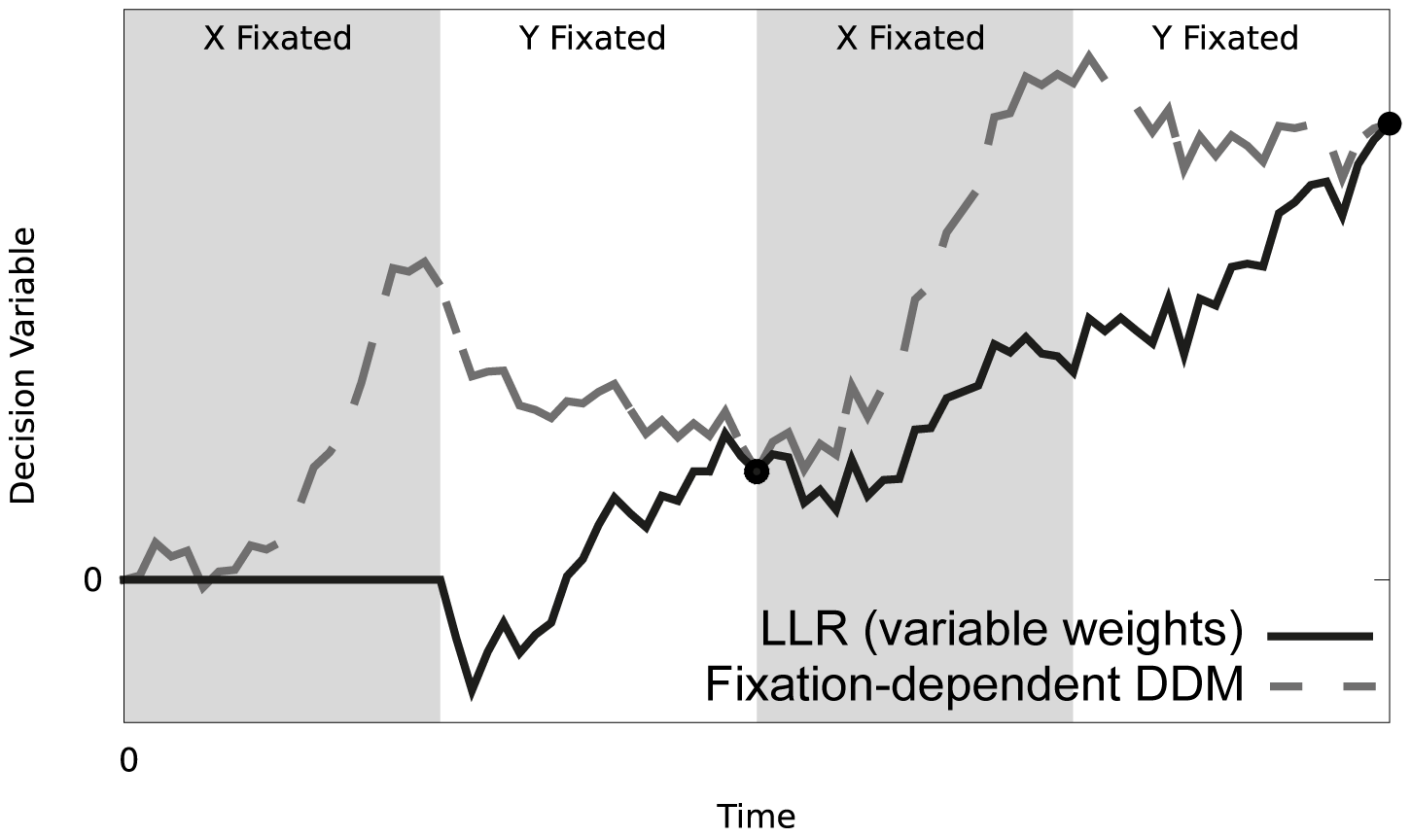
Temporal evolution of the log-likelihood ratio (Equation 5 – thick black line). We also show a simplified decision variable described later in the text (dashed dark grey line; section ‘Fixation-dependent drift diffusion model’ in the Results). The decision variables were computed on the basis of identical sequences of sensory evidence generated from Gaussian distributions with means *μ_x_* = 4 and *μ_y_* = 1, and equal standard deviations *σ_x_* = *σ_y_* = 2.0. The sampling strategy used for both variables is identical and can be inferred from the background of the figure with grey indicating alternative *X* is fixated and white alternative *Y*, each block of fixation is equally sized. For easier comparison the simplified decision variable is scaled by *c*(1-*q*), where *c* is the scaling factor in front of square bracket in Equation 6. The simplified decision variable (Equation 10) in this example assumes time is allocated equally to the two sources (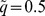) and the points marked by black circles on the figure indicate the times when this assumption holds (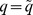).

The weights on the two evidence sums from both alternatives (ratios before the summation signs in Equation 5) vary as samples are drawn from either alternative. This variability in the weight applied to the accumulation of evidence ensures that when one source of information has yet to be sampled, i.e. either 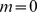 or 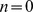, the log-likelihood ratio remains fixed at its initial value of 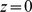. To illustrate this property, the black curve in Figure 2 shows how *z* changes during an illustrative comparative decision, in which positive values indicate a greater amount of evidence for 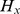 – please note that the curve remains at 0 until the second source is sampled.

### Optimal sampling allocation

We now consider how to allocate the available time between the two information sources to maximize the accuracy in decision tasks with a fixed time limit. The log-likelihood ratio from Equation 5 may be reformulated in terms of the total sampling time 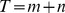 and the portion of this sampling time allocated to alternative 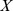, which we denote 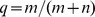. We can then rewrite Equation 5 as:
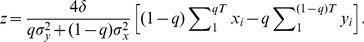
(6)Using the expected value of the log-likelihood ratio at decision time, we can calculate the expected error rate (see Materials S1, section 2.1) for a given sampling strategy (i.e. value of 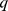):
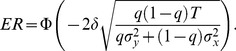
(7)In Equation 7, Φ denotes the standard normal cumulative distribution function. The portion of sampling allocated to alternative *X* that minimises the error rate, can be computed by finding *q* for which *dER/dq* = 0 (see Materials S1, section 2.2 for details). We find that, in order to minimize the expected error rate, the decision maker should divide their sampling time between the two targets such that
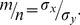
(8)Thus, we can state simply that the optimal allocation strategy is to allocate the available sampling time between the two sources such that each one is sampled for a period of time proportional to its standard deviation. Therefore the ideal observer would spend longer sampling the less certain (or noisier) source.

### Optimal number of switches

**Figure 3:**
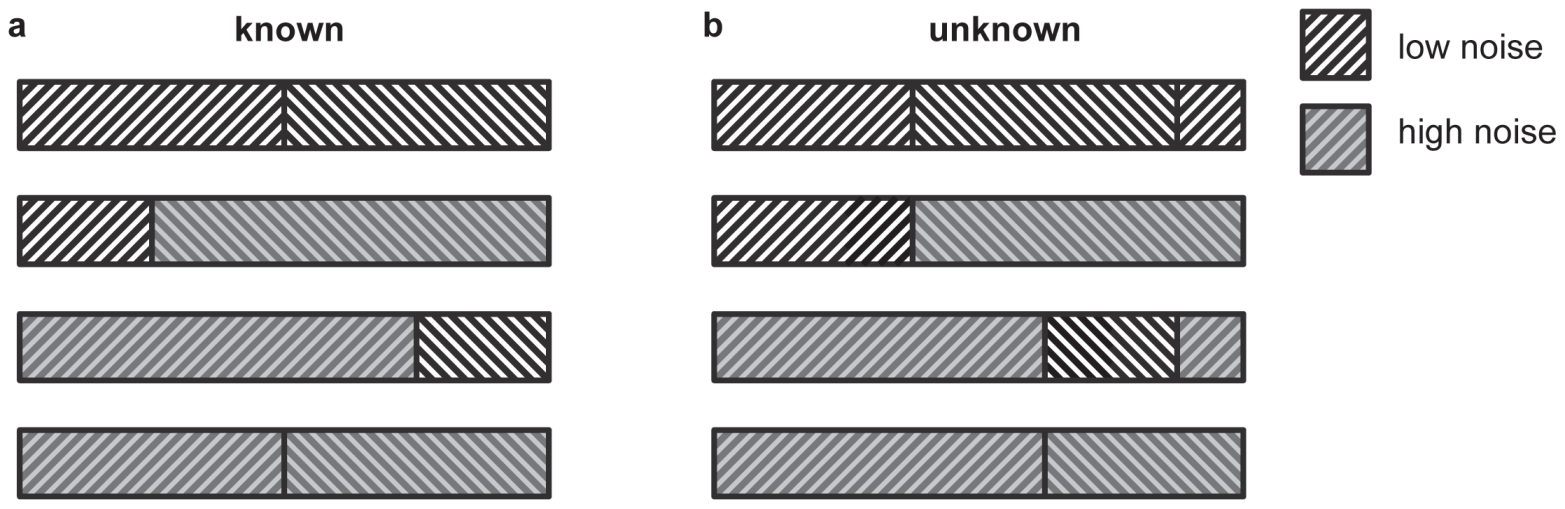
Illustration of optimal switch times in the (a) known and (b) unknown conditions. Each elongated rectangle represents the total viewing duration. Segments with two different orientations of patterns represent the epochs spent viewing the two stimuli. High contrast segments correspond to a low noise source, while low contrast segments correspond to a high noise source. The four rows in each panel correspond to four possible combinations of noise in the two sources.

The decision problem analysed here requires the observer to sample both sources of information, necessitating at least one switch during the course of each trial. Switching frequently entails an energetic and/or temporal cost. For instance, in the particular task outlined below observers make saccades between two stimuli, which results in a period of strongly reduced visual sensitivity [6], [7], [8]. In our mathematical framework, switching between alternatives incurs a temporal cost by reducing the available sampling time *T* in proportion to the number of switches. According to Equation 7, reducing *T* increases the error rate (because Φ is a monotonic function; see Materials S1, section 3 for further details). Therefore, when observers have advance knowledge of the noise levels of the two sources of information, the optimal strategy is to make just the one switch to maximise the time available for sampling the evidence. Figure 3a illustrates schematically the optimal allocation when the observer knows the noise level of both sources in advance.

However, in many natural situations the observers do not know the noise level of the two sources before sampling them. Without prior knowledge multiple switches can, under certain circumstances, be adaptive. Consider a scenario in which there are just two possible noise levels (as in our experiment described below). There are 4 possible combinations of noise level: a low or high noise first source coupled with a low or high noise second source. Figure 3b illustrates the optimal allocation when the observer does not know the noise level of the sources in advance, but only learns of their quality upon sampling them. When the observer does not know the noise level of the second source, say *σ_y_*, while observing the first, the timing of the first switch cannot depend on *σ_y_*. Therefore, the first switch times are the same in the top two cases in Figure 3b. In Materials S1 (section 4.1) we show that the optimal time for the first switch when *σ_y_* is unknown lies in between the two optimal switch times when *σ_y_* is known. For example, the first switch time in the two top cases in Figure 3b lies between the switch times in the two top cases in Figure 3a. When the initial switch has been made and the second source has been sampled, the observer knows the noise level of both patterns. If the second source is of good quality so that not all the remaining time is needed to estimate its mean with sufficient precision, the observer may decide to switch back and collect more information from the first source (first and third cases in Figure 3b). In Materials S1 (section 4.2), we further show that the magnitude of the switching cost influences the extent to which additional switches can improve accuracy.

### Active sampling with gaze

**Figure 4:**
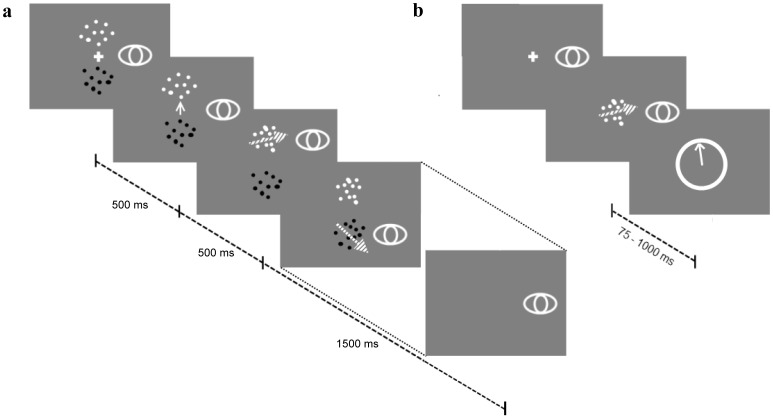
The trial sequences of the presented experiments. (a) Comparative two-alternative forced choice decision task. During each trial observers are presented with two stationary RDKs with centres located above and below the centre point of the screen on the vertical axis. The activation of the RDKs was gaze contingent, with a given RDK activated once the observer's gaze fell within the appropriate region of the display. The task was to identify the target pattern: the one whose signal direction was further clockwise through the short angle. RDKs could consist of either black or white dots in both the full and no prior knowledge conditions but the conditions differed in whether the dot polarity was linked to a noise level. A trial started with a preview period of 1000 ms in which observers could process the polarity of the patterns and were instructed by an arrow (appearing 500 ms after trial onset) which pattern to view first. Following the preview, they were free to actively sample the patterns over a period of 1500 ms and subsequently gave their response once both RDKs were extinguished. They were instructed that they must look at each pattern at least once in the course of a trial but other than that, they could switch between patterns in any way they wanted. The figure shows a trial in which the observer was instructed to view the upper pattern first and made only one switch. The eye indicates the vertical eye position, the hatched arrows the signal direction of motion of the moving pattern. The figure is not to scale. (b) Single pattern direction estimation task. Observers viewed a single pattern between 75 and 1000 ms. Once the RDK had offset, they indicated their estimate of the signal direction by moving an arrow to this direction with a mouse and clicking.

To assess how humans allocate sampling time in this type of decision problem, we performed an experiment in which observers had to judge which of two RDKs translated in a more clockwise direction. Figure 4 illustrates the paradigm in detail. The comparative nature of this judgement meant that both sources of information had to be inspected in order to come to a decision. The dots moved only when fixated, so that only one pattern could be sampled at a particular point in time. Compared to the standard motion discrimination task with just a single source of evidence, the observer not only had to accumulate evidence from each individual source in order to estimate its direction of motion, but also had to decide at each point in time which information source to sample (i.e., take more samples from the current source or switch to the other source?).

Eight observers viewed two RDKs, with either low noise (24% coherence) or high noise (12% coherence) over a period of 1500 ms. Each observer was tested in two conditions (the order of which was counter-balanced). In the ‘known’ condition the luminance of the dots accurately mapped onto noise levels (e.g., black dots represent 24% coherence, while white dots represent 12% coherence). Since participants could see luminance of the dots before motion onset, they had a perfectly reliable cue to the quality of both patterns before actively sampling them. In the ‘unknown’ condition, the luminance of the RDKs was assigned randomly from trial-to-trial.

The optimal observer model developed above makes the following three main predictions for this task: (i) On the trials in which the two patterns had different levels of coherence, the participants should spend more time looking at the noisier stimulus. (ii) The ratio of durations spent looking at the two stimuli should be equal to the ratio of noise levels in the stimuli. (iii) Participants should produce more switches in the unknown than the known condition, in particular when the second stimulus they sample has a high coherence. In the next section we briefly report the accuracy of participants. In the following three sections we compare the above predictions with the experimental data, and report other effects present in the data that were not predicted by the model.

### Perceptual performance

Table 1 shows the behavioural performance data (discrimination accuracy) as a function of the noise in the two information sources, for both levels of prior knowledge. Accuracy averaged across observers ranged from 70% correct (for two low coherence patterns) to 84% (for two high coherence patterns). Performance in conditions with one high and one low coherence pattern, was in between the two extremes, at 76% correct. We combined the data from the two mixed coherence conditions and ran a 3×2 repeated measures ANOVA with pattern coherence (low – mixed – high) and prior knowledge (known – unknown) as factors. The variation in coherence between the patterns was clearly sufficient to generate variations in discrimination accuracy: there was a main effect of pattern coherence, *F*(2, 14) = 39.87, *p*<.001. Prior knowledge did not affect discrimination accuracy, *F*(1, 7) = 1.17, *p* = .32, nor did it interact with coherence, *F*(2, 14) = 0.53, *p* = .60.

**Table 1:** Discrimination accuracy for the different combinations of pattern noise and prior knowledge.

		pattern 2 coherence			
		known		unknown	
		low	high	low	high
**pattern 1 coherence**	Low	0.7	0.76	0.71	0.78
	High	0.76	0.83	0.76	0.85

Values are proportion correct averaged over 8 observers. The within-subject standard error of the mean was 0.01 in all eight conditions.

### Gaze time

To test whether participants spent longer sampling the noisier pattern, we computed the total amount of time spent looking at the pattern that was cued first, 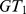. This time includes re-fixations on trials in which observers switched more than once. This gaze time measure is appropriate because the overall presentation time was fixed at 1.5 s, so that the gaze time on the two patterns was not independent: the longer the observer sampled pattern 1, the less time was available for pattern 2 and vice versa. By only taking the gaze time on the first pattern, we ensured that our outcome variable was independent across the different conditions. We converted gaze time into a proportion of the overall sampling time: 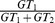. Note that the available sampling time was less than the presentation time due to (i) the latency of the initial saccade to the first pattern, and (ii) the movement time associated with switches from one pattern to another. The former introduced only a minimal delay of 14 ms (average of mean latency across observers), because the interval between cue onset and start of the test period was held constant. As a result, observers frequently anticipated the offset of the cue (start of the test period) and, on average, fixated the first pattern around the time of cue offset. The average saccade duration was ∼50 ms, which is a lower bound on the switch cost (pre- and post-saccadic suppression would prolong this period). For each observer, the proportion of time spent on the first pattern was averaged across trials in a given experimental condition.

**Figure 5:**
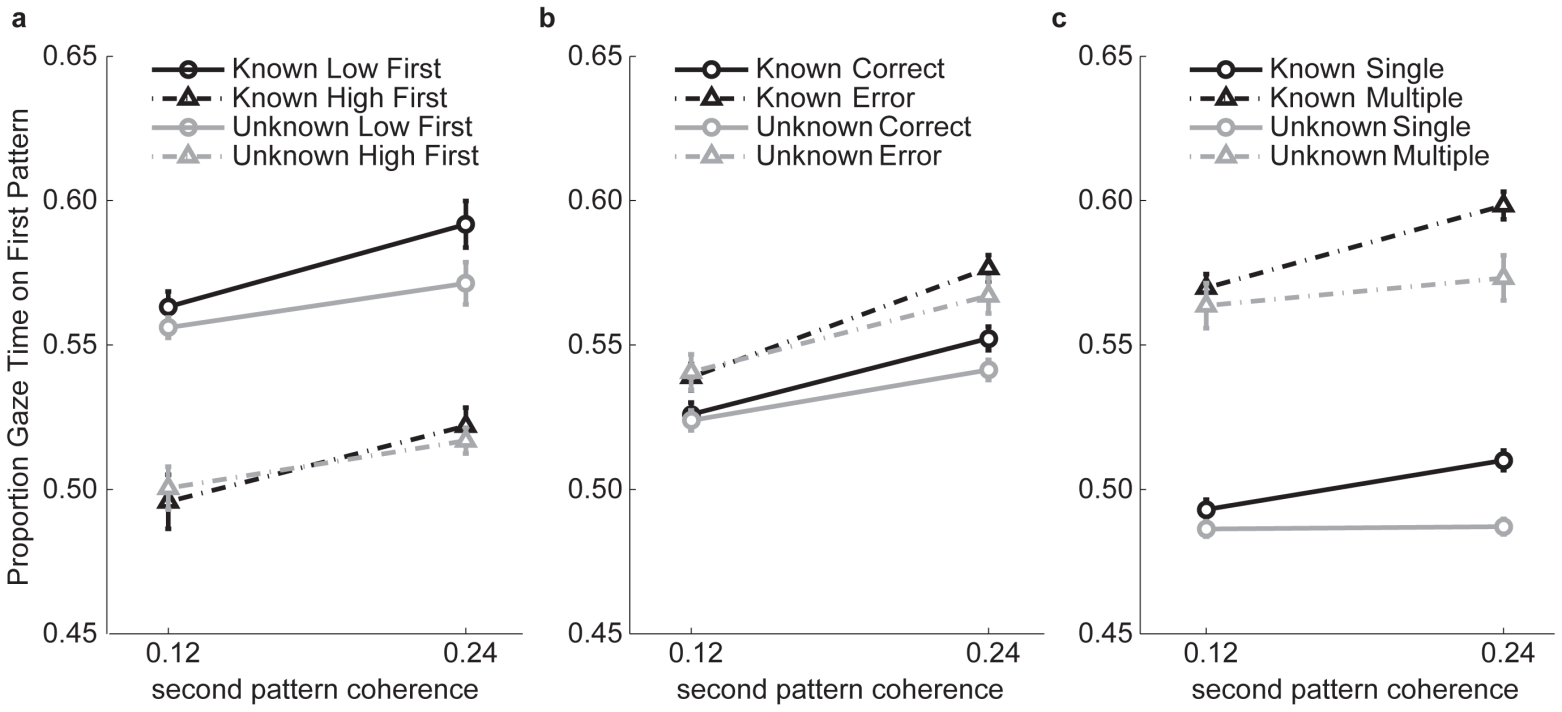
Sampling allocation as measured by the gaze time on the first pattern. Gaze time on the first pattern includes all re-fixations and is expressed as a proportion of the overall gaze time on both patterns. In other words, this variable corresponds to the proportion of available sampling time (excluding the initial saccade latency and the duration of any subsequent movements) spent on the pattern that was cued first. Data are averaged over eight observers and the error bars display within-subject standard error [23]. (a) Gaze time contingent on the coherence of the first and second patterns. (b) Gaze time contingent on the accuracy of the perceptual decision. (c) Gaze time contingent on the number of switches.

Figure 5a shows the sampling allocation for all trials, regardless of the accuracy of the perceptual decision (average of the subject means). Clearly the coherence of the first pattern had a substantial effect on the time it was viewed, with a low coherence pattern being viewed considerably longer than a high coherence pattern, *F*(1,7) = 38.1, *p*<.001. This effect would be expected if observers simply sampled the first pattern until (s)he felt sufficiently certain about the direction of that pattern, and then spent the remaining time in the trial sampling the second pattern. A more critical and interesting aspect of sampling behaviour is whether the amount of time spent on the first pattern depends on the coherence of the *second* pattern. The data in Figure 5a do indeed show a modulation of gaze time by the noise level of the second pattern. When the second pattern coherence was high, more time was spent on the first pattern, *F*(1,7) = 9.35, *p* = .02, as predicted by the model. It is worth noting that the effect of first pattern coherence was larger than that of second pattern coherence, a form of hyper-sensitivity to the first source of information encountered. As a consequence of this hyper-sensitivity, one model prediction is obviously incorrect: when the two patterns have the same coherence, the amount of time allocated to the patterns should be equal (and half the available time). However, observers viewed the low coherence first pattern longer than a high coherence first pattern, even when they had a perfectly reliable cue telling them that the second pattern had the same coherence (i.e. in the known condition).

When prior knowledge about the noise levels of the two information sources is available, observers have the opportunity to adjust the time spent sampling the first pattern depending on the quality of the second pattern *already on the first fixation*. When no prior information is available, the quality of the second pattern is only known once the observer has started sampling it. As such, we might expect a stronger effect of the second pattern coherence when prior knowledge is available. Indeed, there was a significant interaction between the level of knowledge (known vs unknown) and the second coherence, *F*(1,7) = 21.67, *p* = .002. Evidently, observers used prior information to adjust their sampling allocation (see also Figure 5c and text below)

In the remaining panels of Figure 5, we demonstrate the pattern of sampling allocation contingent upon the accuracy of the perceptual decision (5b) and the number of switches between the two sources of information (5c). In Figure 5b we pooled the data across the different noise levels of the first pattern, but split the data depending on the accuracy of the perceptual decision. The most salient aspect of this plot is that on error trials, gaze was fixed longer on the first pattern compared to correct trials. It is possible that observers were more likely to make an error if they switched too late from the first source and did not leave sufficient time for the analysis of the second pattern. Alternatively, it may be that, due to the stochastic nature of the stimulus, on some trials the first pattern happened to be more difficult to encode. The greater difficulty would increase the probability of an error decision, with subjects (appropriately) spending longer on the difficult first pattern. We cannot distinguish between these alternative explanations with the current data set.

In Figure 5c we again pooled across the different noise levels of the first pattern, and included both correct and error trials, but split the data on the basis of switching frequency. It is immediately obvious that observers spent more time on the first pattern when they switched more than once. Indeed, of the multiple switch trials, the majority of trials (∼75%) were those in which observers switched back to the first pattern and stayed there until the end of the trial. Another obvious effect is that when observers switched only once, the coherence of the second pattern could not influence the time spent on the first pattern in the unknown condition (solid grey line). In the known condition, however, the coherence of the second pattern influenced the fixation duration on the first pattern on single switch trials (solid black line), again underlining the use of prior information by our observers. Finally, it is worth pointing out that when observers switched only once, they allocated time nearly equally to both sources of information, suggesting that observers had an accurate representation of the overall amount of time available and used this knowledge to adjust the timing of a single switch. Indeed, for these trials, observers spent less than half the available time on a high coherence first pattern; when the first pattern had a low coherence, observers spent more than half the available time on that source (data not shown).

### Sensory variability and gaze time

To test the second, quantitative prediction on the dependence of gaze time on the pattern noise levels, we would need to know the standard deviations of the evidence generated by the two sources of information (the 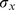 and 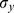 in Equation 8 above). However, the nature of the RDK stimulus is such that we cannot directly map coherence onto estimates of the variability of the information sources. All the observer can know about 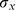 and 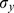 is based on the precision of his/her own direction estimates: a low coherence patch will generally elicit less precise direction estimates compared to a high coherence patch. A complicating factor is that the precision of an observer's direction estimate will depend not just on the external noise in the stimulus, but also on the intrinsic noise of the neural mechanisms that are involved in processing that stimulus [24]. Nevertheless, we took the view that internal noise could not have been so large as to completely swamp the influence of the external noise on the observers' direction estimates; otherwise, discrimination performance would have been equal in the conditions with two low or two high coherence patterns (see Table 1). As such, it is informative to examine whether and to what extent the allocation of gaze time could be predicted from the directional (un)certainty.

**Figure 6:**
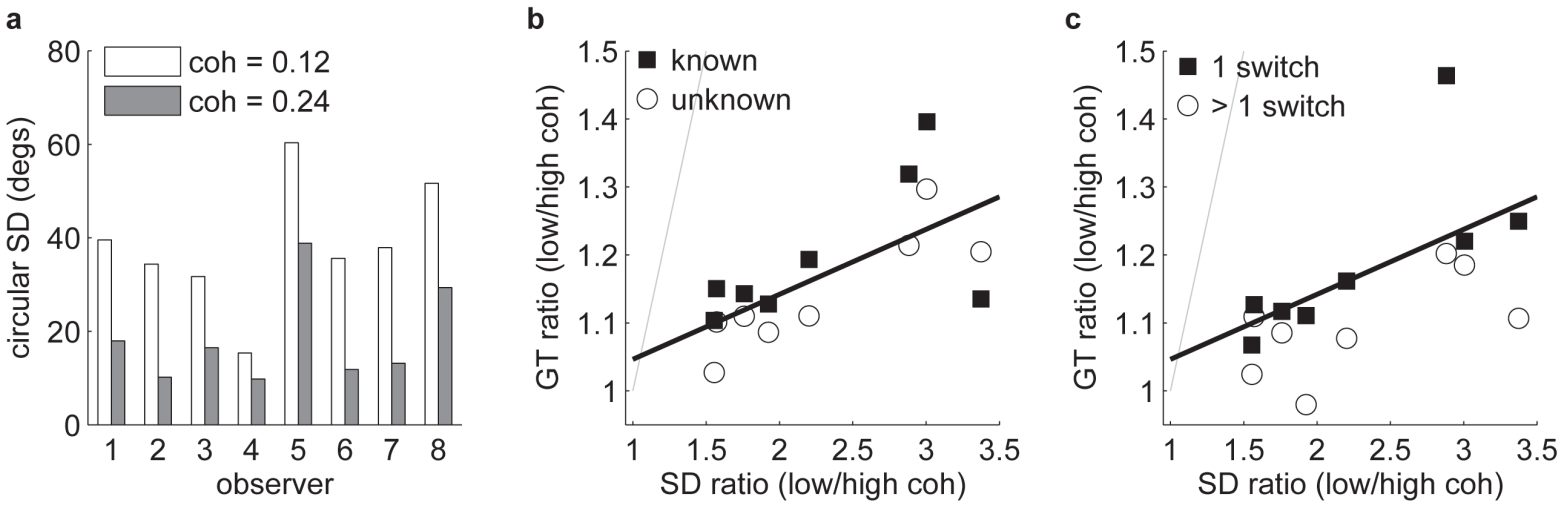
Sensory uncertainty estimates and their relation to gaze time. (a) Standard deviation of the angular errors (angular deviation metric; see Methods for details) in the single pattern direction estimation task. Patterns were viewed for 750 ms (close to the average gaze time on the first pattern in the main experiment). (b) Gaze time ratio of low and high coherence first patterns (from trials in which the coherence of the two patterns differed) versus the ratio of the standard deviation of the single pattern direction estimates. Data are shown separately for known and unknown conditions (*N* = 8 in both conditions). The thick black line shows linear regression on the data averaged across the two levels of prior knowledge. The thin grey line shows the identity correspondence. (c) Same as panel b, but with the gaze time data pooled across prior knowledge and split depending on the number of switches. The thick black line shows the same regression as in (b).

To measure directional uncertainty, observers were shown a single low or high noise pattern and were asked to indicate the direction of coherent motion by rotating a dial with the mouse cursor. The true motion direction was uniformly sampled around the clock in integer intervals. For each direction judgement, we calculated the difference between the actual and perceived direction, with a positive sign given to clockwise deviations. Across a number of repetitions at a given coherence, we calculated the circular standard deviation (angular deviation in [25]). We estimated the variability in this way for a number of different viewing durations (see Methods; data not shown), but for the purpose of the present analysis we selected a viewing duration of 750 ms as the one that came closest to average gaze time on pattern 1. Figure 6a shows these standard deviation estimates for each individual observer and for the two different levels of coherence. As expected, uncertainty was much greater for the low coherence pattern compared to the high coherence pattern for every individual observer. These variability estimates served as proxies for the generative 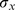 and 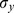 in the following analysis.

The model prediction given by Equation 8 is straightforward: the ratio of sampling time allocated to the two patterns should match the ratio of the standard deviation of the evidence provided by the two patterns. Of particular interest are of course the trials in which the quality of the two information sources differed. We selected only these trials and computed the ratio of the gaze time on the low coherence pattern (in low – high coherence trials) to the gaze time on the high coherence pattern (in high – low coherence trials). In this way, we ensured that the gaze time estimates that make up the ratio were independent. Figure 6b plots the low/high coherence gaze time ratio against the low/high coherence ratio of the perceptual direction judgements, separately for the two levels of prior knowledge.

Several features are noteworthy. First, all the data lie below the identity line (thin grey line), indicating that gaze time was much less differentiated than the sensory noise would dictate. The existence of internal noise does not explain this departure from the model prediction: internal noise – more specifically stimulus-independent internal noise – would push the ratio of the sensory variability closer to one (effectively adding a constant to the squared numerator and denominator on the right side of Equation 8). In other words, for a noiseless observer limited only by the external noise in the stimulus, the data points would lie even further to the right. Second, the gaze time ratio was consistently lower in the unknown condition compared to the known condition (in 7/8 observers). This finding underlines that prior knowledge allowed for a stronger dependence of the sampling strategy on the quality of the information sources, as already shown above (Figure 5). Third, while the strong identity prediction of the model did not hold, there clearly was a strong relation between the precision of the direction estimates and gaze time. The thick black line corresponds to the regression line derived from all the data (averaged across known and unknown conditions). The slope is significantly greater than 0 (*p* = .04) and explains over half the variance, *R* ^2^ = .55.

Figure 6c shows the relation between sensory variability and gaze time contingent upon the number of switches made by the observer (pooled across prior knowledge). The thick black line is the same regression function from panel b, based on all the data taken together. The regression line is closer to the single switch trial data for the simple reason that these represent the majority of the trials. However, it is notable that the multiple switch trial data points consistently lie below the single switch observations (for all 8 observers). That is, gaze time was less sensitive to the quality of the information when observers switched more than once. One possible explanation is that additional switches may be triggered if on some trials observers fail to adapt their very first fixation to the quality of the information. On such trials, any difference in the gaze times on low and high coherence first patterns may only be engendered during the third fixation (or any subsequent fixations on the first pattern), for which much less time is available. Inevitably then, the gaze time ratio will be closer to unity.

### Switching Frequency

**Figure 7:**
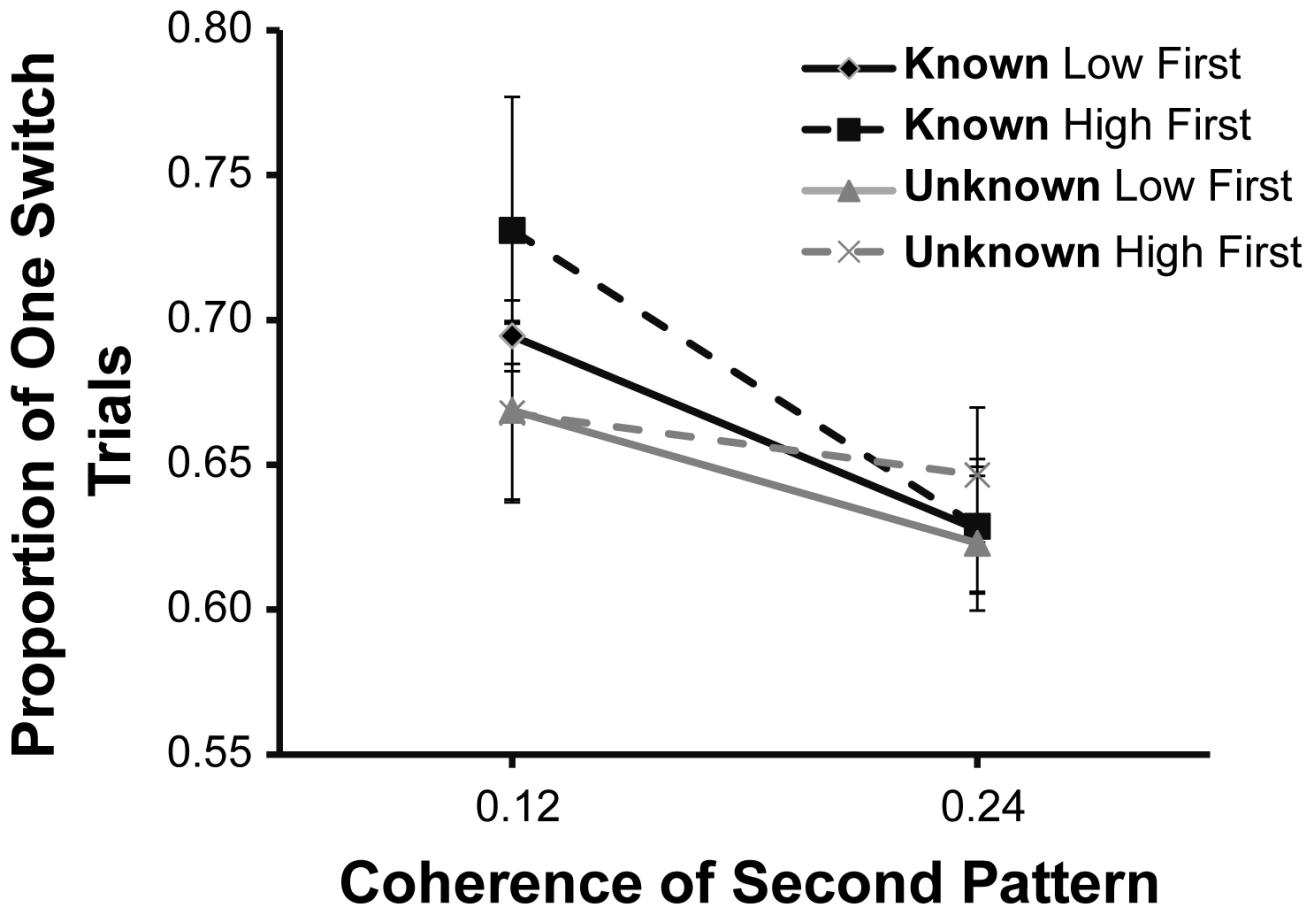
The proportion of trials on which a single switch between the RDKs occurred. Data are averaged over 8 observers. The error bars display within-subject standard error.

Finally, we turn to a description of observers' switching behaviour. Recall the strong model prediction that observers should switch only once in the known condition. It may be beneficial to switch more than once in the unknown condition, when the second pattern had a high coherence. Figure 7 shows the proportion of single switch trials. Clearly, observers did regularly switch more than once, even when prior knowledge was available about the quality of the two sources, which is inconsistent with the prediction of the model. The negatively sloping lines indicate a dependence of the switching frequency on the coherence of the second pattern. That is, observers were more likely to switch more than once when the coherence of the second pattern was high, *F*(1,7) = 10.9, *p* = .01. This result is in line with the prediction of the model. There was an interaction between prior knowledge and second pattern coherence, *F*(1,7) = 7.93, *p* = .03. Figure 7 suggests that this interaction is due to an increased propensity to make only a single switch when prior knowledge was available and the second pattern had a low coherence. It is tempting to speculate that when prior knowledge was available, observers were better able to optimise a single switch point. Setting an appropriate switch point may be especially important when the quality of the second source is relatively poor. Arguably, however, choosing the right switch point is most important when *both* patterns have a low coherence. There is no evidence that observers made fewest switches on these particular trials.

### Fixation-dependent drift diffusion model

In the Introduction, we noted the recent development of a model to account for decision-making in a specific instance of the more general decision problem we have considered in this article [21]. Krajbich and colleagues studied choice between two consumer products, which were inspected sequentially. The two options were assumed to have equal noise and the decisions were terminated by the observers themselves, rather than after a fixed period of time. Given the obvious relation to the more general problem considered here, we examined the relation between their model and the ideal observer developed in this study.

We start by simplifying the decision variable described in Equation 6. As explained before, in tasks with fixed time available to form a decision and a prior assumption that both hypotheses are equally likely to be true, the optimal observer chooses an alternative on the basis of the sign of the final log-likelihood ratio. Therefore, Equation 6 can be simplified by ignoring the scaling factor in front of the square bracket; as this scaling factor is positive it does not change the sign of decision variable and the choices made by the model. Furthermore, we divide the decision variable by 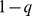, which again does not change its sign. The resulting decision variable is now:
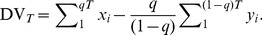
(9)In Equation 9, the evidence sampled from *Y* is weighted by a factor which depends on the relative time spent viewing the two stimuli. Such relative weighting is not present in the standard diffusion model that describes choice between two alternatives for which evidence comes in simultaneously. The comparative nature of the current problem results in the relative weights on the two sources of evidence changing every time one particular source is sampled. Such continuous adjustment of the weights may be difficult to achieve computationally in a real biological system.

Therefore, we consider a simplified procedure for updating the decision variable that does not involve changing the weights of accumulated evidence over time. Under some specific circumstances, this procedure still results in exactly the same value of decision variable at the end of the available time *T* and hence the same choice. Suppose the decision-maker aims to spend a certain fraction of time on each alternative. In practice this may be difficult to achieve exactly, but it seems plausible that the observer has learned how much time is available overall, and how much time is typically needed to get a reasonable estimate of the stimulus property under consideration (e.g. motion direction). We denote the target fraction of samples 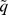. For example, observers might aim to spend half the available time on each option, so that 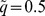. To update Equation 9, the observer now uses the target fraction 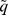 instead of the “true”, evolving fraction 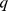. As a result, the decision variable is now updated as follows:
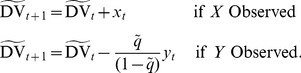
(10)Note that the decision variables of Equations 9 and 10 are equal if 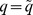. Thus if the actual allocation of samples matches the target fraction, then at decision time *T* this simplified procedure results in exactly the same decision variable as the ideal solution.

To illustrate the relationship between a decision variable with variable weights (Equation 5) and one with fixed weights (Equation 10), we simulated runs of the two decision variables with identical sequences of samples used for each (Figure 2). In the simulations of the simplified decision variable, the weighting was based on a target of sampling both alternatives equally (i.e. 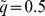) and the points marked by black circles on the figure indicate the times when this assumption holds (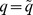). At each of these points the simplified decision variable is equal to the log-likelihood ratio (marked by filled circles). At all other points the true sampling allocation *q* differs from the assumed terminal value 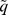 and the simplified decision variable differs from the log-likelihood. Interestingly, the deviation is such that for these periods the decision variable drifts toward the fixated alternative.

If the observer using Equation 10 allocated samples to the two alternatives differently to the target fraction, so that 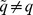 at the end of sampling time, then the error rate increases (see Materials S1, section 5). When 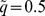, the decision maker will be biased towards choosing the alternative sampled for longer. Such an influence of gaze time was demonstrated in the “consumer choice” paradigm of Krajbich et al [21], and was the basis for the development of their fixation-dependent drift diffusion model. In fact, for the specific case illustrated here with 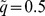, the weighting factor in the bottom of Equation 6 equals 1. The resulting decision process reduces to that proposed by Krajbich et al. [21]. In our data there was no such bias for observers to choose the pattern that had been sampled for longer. In the equal coherence conditions, the chosen pattern was sampled for 51.3% of the available time (averaged across observers). In the unequal coherence conditions, the chosen pattern was sampled for 51.8%. The small deviations from the equal sampling allocation amounted to ∼40 ms, but the proportional gaze times did not differ reliably from 0.5 (one-sample t-tests, *p*>.1).

Note that Equation 10 describes the temporal evolution of the decision variable regardless of whether the observer manages to match the target fraction 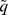. In our view, it is not unreasonable to assume that, after some experience on the decision task, observers have some idea of how long they should spend sampling each source of information (in the absence of any information about the upcoming stimulus). The adoption of a certain value for 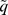 may be seen as a heuristic that, while not optimal, typically results in a decision variable that is reasonably close to optimal to enable satisfactory performance. With prior knowledge about the quality of the two information sources, it becomes – in principle – possible to set a more appropriate value for 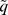. Indeed, we have shown that on single switch trials, fixation duration on the first source is adjusted to the quality of the second, un-seen source under such conditions (Figure 5c). On any one trial, however, there will be other determinants of gaze time that influence how close the actual allocation of time matches the target allocation. Some of these other influences are discussed below.

## Discussion

In this paper we studied a challenging decision problem in which information from two different sources is sampled in order to come to some overall decision about the “state” of the visual world (for a recent, related development in the context of visual search, see [26]). In the majority of studies on the neuroscience of (perceptual) decision-making, a binary decision is made on the basis of a single source of information that simultaneously provides evidence for both alternatives [9], [10]. The problem introduced in this article goes beyond this simple situation in that (i) there is limited time to sample different sources of information or options; (ii) sources can only be sampled one at a time; (iii) the quality of different sources may vary; (iv) the quality of the sources may not be known beforehand; and (v) switching between sources incurs a temporal cost. While the specific instantiation of the decision problem was perceptual in nature, these characteristic features of the problem are shared with more complex situations such as economic decisions (e.g., choosing a consumer product based on sampling user reviews), and different forms of animal behaviour [27] (e.g., foraging in patchy environments; mate choice).

Given the novelty of the problem under consideration, as a starting point we identified the optimal solution or “ideal observer” [28]. We then placed human observers in a similar decision situation and compared their behaviour with that of the optimal observer. Finally, we developed a simplified, non-optimal observer that does not require continuous adjustment of the weights applied to evidence from the two sources. We showed that this simplified model contains the fixation-dependent drift-diffusion model [21] as a special case. This model has been developed recently to account for choice behaviour in a similar decision problem that involved sampling non-stochastic options in serial, under the assumption that both options were of equal quality. In the remainder of the Discussion, we focus on the comparison between optimal and human observers. In particular, departures from the normative solution should tell us something about the constraints faced by a real biological system in the solution to this type of decision problem.

The most trivial prediction of the model is that noisier sources of information should be sampled for longer and human observers did exactly that (Figure 5a). A more specific prediction is that the ratio of gaze time on low and high coherence patterns maps onto the ratio of the standard deviation of these sources of information. We did not have direct access to the stimulus noise, but we inferred the ratio of the sensory variability from observers' uncertainty about the direction of individual motion patterns (measured separately). While these data were not in line with the predicted identity correspondence, there was a clear correlation between gaze time and the precision of the direction estimates (Figure 6b). Furthermore, the optimal observer predicts that a second switch (back to the first pattern) will be more likely if the *second* pattern is of good quality, so that not all the remaining time is needed for its analysis. The downward slopes in Figure 7 are consistent with this prediction.

An important question concerns *how* observers know the quality of a pattern or their direction estimate. For the patterns themselves, with only two levels of noise it is relatively easy to rapidly classify a pattern as ‘low noise’ or ‘high noise’. Observers may simply know that noisier patterns result in less certain direction estimates, or they may have learned a more precise mapping during the experiment itself (or, indeed, during the preliminary threshold estimation phase of the study). In terms of an *online* mechanism that estimates the uncertainty while viewing a particular pattern, it may be possible to monitor the stability of an internal direction estimate over time. For example, large fluctuations from time *t* to time *t+n* would indicate large variability. The simplest mechanism would involve a simple comparison between two (successive) time points; more complex mechanisms would involve computing the second-order statistics over a number of direction estimates from a larger temporal window.

It is clear that human behaviour departed from the norm in several ways. First, even when given prior information, observers often made more than one switch, whereas the ideal observer would set just a single switch point. At the very least, we might have expected the number of switches to be lower when prior knowledge was available, but no overall reduction in the number of switches was observed (Figure 7). Second, as stated above, for the optimal observer the ratio of sampling or gaze times matches the ratio of variability of the information sources. In our data, however, the modulation of gaze time was much less than what would be expected from the precision of the direction estimates (Figure 6b). Third, the model predicts that when the noise of the two information sources is equal, gaze time should be equal regardless of coherence when the noise levels are known beforehand. That is, gaze time on the first pattern in a trial with two low coherence patterns should be equal to the gaze time on the first pattern in a trial with two high coherence patterns. Indeed, the model makes a stronger prediction that time should be allocated equally to the two patterns within a trial in both of these conditions. Neither prediction was supported in our data: gaze time was longer on the low coherence pattern compared to the high coherence pattern in the equal coherence trials (Figure 5a). In addition, overall gaze time on the first pattern was generally greater than half the available time. The latter result is mostly due to re-fixations: on trials with just a single switch, gaze time on both patterns was much more equally distributed (Figure 5c).

These departures from the optimal observer are not surprising: the optimal observer is only driven by the quality of the information on the current trial and has perfect memory of all the information sampled. With prior knowledge, the optimal observer switches only once, at a time that maximises the amount of information gained from the two patterns. Without prior knowledge, the optimal observer makes allowances for the un-known quality of the second pattern. When the second pattern turns out to be relatively easy to process, a further switch back to the first pattern may occur. It is clear that our human observers are influenced by other factors that are not directly linked to the quality of the current stimulus. We will discuss some of these factors separately for switch frequency and fixation timing.

### Switch frequency

What triggers multiple switches in a trial? In particular, why would observers go back to a pattern they have already sampled? In the absence of prior knowledge, we described how it may be advantageous to switch back if it turns out that the first switch left more time than necessary for the analysis of the second pattern. However, our observers switched back almost equally often when prior information was available. It is of course plausible that the efficacy with which that information is used fluctuates from trial-to-trial. Furthermore, even when prior information is used appropriately, the stochastic nature of the stimulus may make one of the patterns more difficult to identify than expected, in which case switching back to that pattern is appropriate. It is also possible that observers sometimes switch back as a checking operation in order to verify a preliminary decision, provided sufficient time is available. Moreover, noise or errors in the timing of the first switch may necessitate a further switch back to further process the pattern that was processed too briefly the first time around.

Finally, it is possible that when the observer switches to the second source, the representation of the first pattern direction degrades, perhaps due to passive decay or interference by the currently sampled stimulus [29], [30]. Decay and interference result in a less certain estimate of the signal direction of the previously sampled pattern [31]. An observer may then switch back to a previously sampled source in order to compensate for this loss of information. The model assumes that each sample contributes equally to the perceptual decision – in other words, that there is perfect memory. This assumption is most likely not valid for human observers. If decay or interference occurs, earlier samples effectively contribute less and the need for further switches becomes more pressing.

### Fixation timing

The factors listed above may influence switching behaviour and thereby gaze time. Of course, gaze time is also directly influenced by how long observers choose to fixate a given pattern. In this domain too, it is likely that human observers are influenced by factors other than the quality of the two patterns. In particular, it is plausible that observers in a task like this develop an overall sense of the total trial duration and the typical amount of time needed to identify the direction of the two patterns with sufficient accuracy. Indeed, on trials with a single switch – especially in the known condition – observers spent approximately half the available time on each pattern (Figure 5c). We take this finding to suggest that observers have a good representation of the overall trial duration and use this knowledge to ensure that approximately sufficient time is available to process both patterns.

In drawing a link between the optimal observer and the fixation-dependent drift diffusion model, we posited exactly such a temporal representation (in the form of the target fraction of samples allocated to a given source, 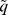). For example, observers may learn that most of the time, they feel reasonably confident about their decision when they switch somewhere near the mid-point of the trial. This strategy may then form the basis of their sampling behaviour, with the specific properties of the stimulus on any given trial only modulating this “default setting”. As a result, any modulation of the sampling allocation by the quality of the information sources would be much more subtle than predicted on the basis of the stimulus qualities alone. Note that some models of eye movement control in reading [32], [33] and scene perception [34] incorporate the idea of a rhythmic timing mechanism that paces movements of the eyes at a rate that is typically sufficient to allow for adequate information uptake during individual fixations [35].

### Is sampling behaviour adaptive?

Given these departures from the norm, it is reasonable to ask whether, and in what sense, sampling behaviour was adaptive at all. Human data did not show a strong relationship between gaze allocation and decision accuracy on a trial-by-trial basis (data not shown). The absence of such a link between sampling and accuracy most likely stems from the carefully titrated difficulty of the discrimination task. That is, we may have set the directional offset at such a level that relatively small variations in sampling time are unlikely to generate drastic modulations in perceptual accuracy. In our view, adaptive sampling means that the time allocated to the available information sources reflects the uncertainty in the *global* task environment, since the quality of *all* information sources is relevant to good task performance.

In this paper, we have reported a number of effects that demonstrate that the quality of multiple sources of information, beyond the currently fixated source, influences the sampling strategy. Even in the unknown condition of the present study, we see an effect of the noise of the second source on the sampling time of the first source. This effect is necessarily mediated by multiple switches. As a result of these switches, the quality of both sources of information influences the sampling time of the first pattern. When we provide the observer with prior knowledge about the quality of both information sources, the quality of the global task environment exerts a more pronounced effect on gaze time, the number of switches, and even the timing of a single switch. We suggest that these strategic adjustments to the quality of information in the global task environment are the hallmark of adaptive sampling.

## Methods

### Participants

Eight observers (5 females; age: 18–25) received money for participation. All had normal vision or vision corrected by contact lenses. Participants provided written consent before testing began, and once more after testing had been completed and they were debriefed. The experimental work was approved by the University of Bristol Faculty of Science Human Research Ethics Committee and complied with the Declaration of Helsinki.

### Stimuli

Stimuli were presented on a 21″ Viewsonic G225fB monitor with 1024×768 resolution at 85 Hz and were generated by custom-written software running in Matlab (The MathWorksLtd.) using PsychToolbox 3.0.8 [36]. The position of one eye (typically the right) was recorded at 1000 Hz using an Eyelink 2000 video-based eye-tracker (SR Research Ltd.).

One RDK consisted of 100 white or black dots (squares of side length 3 pixels ≈7′) which moved within a circular aperture of radius 4 deg on a mid-grey background, giving a mean dot density of 2 dots deg^−2^. The dot patterns moved according to a ‘Brownian Motion’ algorithm [37]: On each frame, a subset of dots were chosen as signal dots and translated in the signal direction and the remaining (noise) dots were given random directions from the interval [0,360), but moved at the same speed. Each dot moved at 6 deg s^−1^, being translated ∼4′ on each frame. RDK animations were independently produced for each trial for each participant. The two coherences (proportions of signal dots) used throughout the experiment were 0.12 and 0.24. In the main experiment, a “standard” direction was chosen randomly from the interval [0,360) and assigned this to either the top or bottom pattern. The other pattern then moved in the standard direction ± the directional offset determined by the preliminary measurement of the directional discrimination threshold.

### Threshold estimation

Participants sat at a desk and viewed the computer screen, constrained by chin and forehead rests, from a distance of 57 cm. After the presentation of the fixation cross for 800 ms, two RDKs of the same coherence were presented sequentially in the centre of the screen for 600 ms each, separated by a 1 second inter-stimulus interval. The participants' task was to identify the target pattern: the one whose signal direction was further clockwise through the short angle (no trials involved the signal directions differing by more than 80 deg). They signalled their decision with a keypad after the second pattern had offset. Trials began automatically after a delay of 750 ms following the response to the previous trial being registered. Observers were given auditory feedback on their performance (high tone – correct, low tone – error).

In order to obtain threshold directions for each level of coherence, two staircases were implemented using the QUEST algorithm [38], [39]. QUEST sequentially updates a posterior probability density function (pdf) of the threshold location based on a psychometric function with specified slope (determined by a pilot study using the method of constants), chance and lapse-rate parameters. It then suggests the angle of the next trial at the median of the posterior pdf. Finally, QUEST estimates the 75% threshold at the mean of the posterior pdf. Staircases were run concurrently, with trials from each staircase randomly intermixed. QUEST's suggestions for the next trial angle were always implemented. The session began with between 30 and 60 practice trials in which participants could familiarise themselves with the paradigm. Each staircase consisted of 80 trials making a threshold estimation block of 160 trials with two scheduled breaks.

The average of the two thresholds was used as the directional offset between the two patterns in the main comparative direction discrimination task. By setting the directional offset in this manner we ensured that: (i) the overall difficulty of the main comparative task was titrated appropriately for each individual observer; and (ii) the variation in coherence did indeed correspond to a variation in the quality of the internal, sensory evidence; that is, with a much larger directional offset, the coherence of the patterns would have mattered much less.

### Single pattern direction estimation

Observers also performed a task in which they viewed single patterns presented at fixation for a variable duration. Six durations (75, 125, 250, 500, 750, 1000 ms) were crossed with the 2 coherences (.12 and .24) to create 12 experimental conditions. A 13th condition consisted of 100% coherence for 1000 ms. Observers viewed the RDK and then indicated their estimate of its signal direction using an onscreen arrow positioned with a mouse (see Figure 4b). For each trial, a mid-grey background was used with the polarity of the dots (black or white) chosen randomly. Each condition was repeated 55 times, for a total of 715 trials, performed over one hour with three breaks.

For each trial, we recorded the angular difference between the true direction of motion and the estimated direction of motion. Inspection of individual observers' errors as a function of the true direction revealed no consistent biases. The circular standard deviation was computed using the ‘CircStat’ toolbox [25] for Matlab (The Mathworks, Inc). The particular metric we opted for was the angular deviation, which can range from 0 to 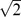 (inclusive) radians [0°–81°]. This particular metric generally gives somewhat lower estimates of the variability than the regular standard deviation that does not take the circularity of the data into account. However, our specific inferences from the analysis presented in Figure 6 do not depend on exactly which metric we select.

### Comparative decision task

A block of trials started with a calibration of the eye tracker, using a nine-point grid. A subsequent validation was used to ensure the consistency of the calibration (mean difference ≤.5 deg). Each trial began with a central fixation cross. Once this was successfully fixated, the experimenter started the trial. Immediately two stationary RDKs appeared, centred 5.8 deg above and below the centre of the screen on the vertical axis. After 500 ms the fixation cross was extinguished and replaced with an arrow pointing either up or down (with equal probability). The arrow was displayed for 500 ms, and its offset was the cue for observers to fixate the cued pattern. Given the fixed duration of the cue, its offset was frequently anticipated by observers; however, the fixated pattern would only start moving after the 500 ms cue period was over. In the ‘known’ condition the luminance (i.e. black or white) of the RDK mapped onto the coherence (the mapping was counterbalanced between participants). In this condition the duration of the cross and arrow gave observers 1 second in which to process the RDK polarities. In the ‘unknown’ condition, the mapping was random; that is, each RDK was randomly drawn with black or white dots.

Participants had to fixate at least 1.8 deg vertically above the screen centre before the upper RDK moved and the same distance below the centre for the lower pattern. This gaze contingency was produced using real-time gaze position information from the eye-tracker, taking into account the vertical component only. Participants were free to actively sample the patterns over a period of 1500 ms. At this point, the patterns disappeared and participants signalled whether the top or bottom pattern moved in a direction that was “more clockwise”. They were instructed that they must look at each pattern at least once in the course of a trial (indeed, the task was impossible without sampling both patterns) but other than that, they could switch between patterns in any way they wanted.

After 10–20 practice trials to familiarise themselves with the main comparative task, participants performed 24 blocks of 64 trials over 4 experimental sessions: 12 blocks in each of the ‘known’ and ‘unknown’ conditions. The ‘known’ and ‘unknown’ conditions were alternated between blocks within a session; the order was counterbalanced across observers. A block consisted of 16 trials for each combination of the two coherence levels. Participants were allowed breaks between blocks. The whole study was performed in six one-hour sessions on different days, in the following order: threshold estimation, comparative decision 1, comparative decision 2, single pattern direction estimation, comparative decision 3, comparative decision 4.

## Supporting Information

Figure S1
**Division of sampling time for varying combinations of high and low noise stimuli for instantaneous switching decision problems.** In Panel (a) the timelines show the division of sampling time between the sources of stimuli for the four possible combinations of high and low noise stimuli with the noise of both sources known a priori. In each timeline we show the simplest single switch strategy, the timing of the switch is indicated by the vertical line, with alternative X observed from time 0 to the switching point and alternative Y observed thereafter until the trial ends at time T. In Panel (b) the timelines show the range of times in which the optimal first switching point lies when the noise level of stimulus Y is unknown. Unlike the known variance case, the exact location of the optimal first switching point depends on not only the two possible noise levels but also depends on the total sampling time available and 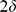, the difference in the response to the stimuli.(TIFF)

Figure S2
**Plots comparing the argument of the DV (blue lines) and LLR (red lines) error functions across the interval of valid sampling strategies [0, 1] under a number of parameterisations of the decision problem.** In each plot the optimal sampling strategy 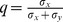
is marked on both the argument and derivative plots. Plots have been generated with 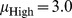, 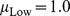, 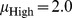, 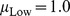 and 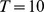. From left to right the variances of the alternatives vary in each of the plots with Figures 2(a) and 2(d) having 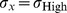
and 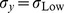, Figures 2(b) and 2(e) having 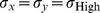, and in Figures 2(c) and 2(f) 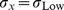 and 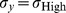. From top to bottom the means of the alternatives vary with Figures 2(a), 2(b) and 2(c) having 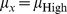 and 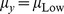 and Figures 2(d), 2(e) and 2(f) having 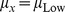 and 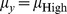. Comparing the DV and LLR plots it can be seen that, as expected, the two values are coincident at 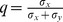. Furthermore, from inspection of the DV argument plot (blue line), it can be seen that as the plot is a straight line, the derivative has a constant value across [0, 1].(TIFF)

Material S1 **Adaptive sampling of information in perceptual decision-making.**
(PDF)
