# Supplementary figures and images for: Adaptive Sampling of Information in Perceptual Decision-Making
Source: PLoS One. 2013 Nov 27;8(11):e78993. doi: 10.1371/journal.pone.0078993 (PMC3842256; doi:10.1371/journal.pone.0078993)

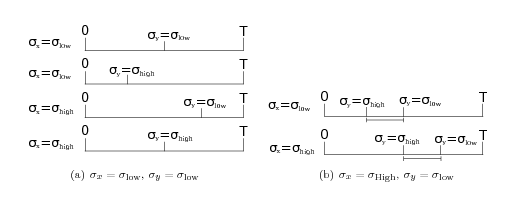

Supplement: Figure S1 — Division of sampling time for varying combinations of high and low noise stimuli for instantaneous switching decision problems. In Panel (a) the timelines show the division of sampling time between the sources of stimuli for the four possible combinations of high and low noise stimuli with the noise of both sources known a priori. In each timeline we show the simplest single switch strategy, the timing of the switch is indicated by the vertical line, with alternative X observed from time 0 to the switching point and alternative Y observed thereafter until the trial ends at time T. In Panel (b) the timelines show the range of times in which the optimal first switching point lies when the noise level of stimulus Y is unknown. Unlike the known variance case, the exact location of the optimal first switching point depends on not only the two possible noise levels but also depends on the total sampling time available and , the difference in the response to the stimuli. (TIFF) [file pone.0078993.s001.tiff]

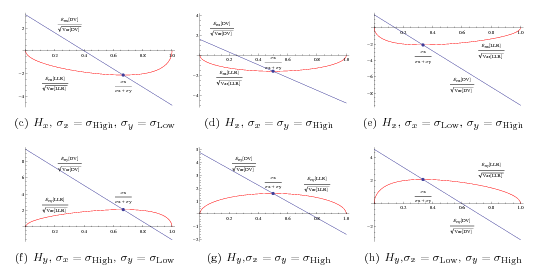

Supplement: Figure S2 — Plots comparing the argument of the DV (blue lines) and LLR (red lines) error functions across the interval of valid sampling strategies [0, 1] under a number of parameterisations of the decision problem. In each plot the optimal sampling strategy is marked on both the argument and derivative plots. Plots have been generated with , , , and . From left to right the variances of the alternatives vary in each of the plots with Figures 2(a) and 2(d) having and , Figures 2(b) and 2(e) having , and in Figures 2(c) and 2(f) and . From top to bottom the means of the alternatives vary with Figures 2(a), 2(b) and 2(c) having and and Figures 2(d), 2(e) and 2(f) having and . Comparing the DV and LLR plots it can be seen that, as expected, the two values are coincident at . Furthermore, from inspection of the DV argument plot (blue line), it can be seen that as the plot is a straight line, the derivative has a constant value across [0, 1]. (TIFF) [file pone.0078993.s002.tiff]
