# Supplementary material for: Adaptive Sampling of Information in Perceptual Decision-Making
Source: PLoS One. 2013 Nov 27;8(11):e78993. doi: 10.1371/journal.pone.0078993 (PMC3842256; doi:10.1371/journal.pone.0078993)
Supplement: Material S1 — Adaptive sampling of information in perceptual decision-making. (PDF) [file pone.0078993.s003.pdf]

Adaptive sampling of information in perceptual decision-making - Materials S1

1 Ideal Observer Derivation

In this section, the derivation of a likelihood-ratio hypothesis test for determining which of two normally distributed populations has the greatest mean is presented. The basis for this test stems from the work of Flehinger et al.[1, 2] who proposed a likelihood ratio hypothesis test for comparing treatments in medical trials in which the data from the two treatments is represented by two normally distributed populations with common variance σ^2. Subsequent contributions made to this body of work[3, 4, 5] provided generalisations to the likelihood ratio test, as well as bounds on the performance achieved in a variety of scenarios. One such generalisation, particularly relevant to the comparative two-alternative decision making problem outlined in the main text, was made by Hayre and Gittins[4] who generalised the hypothesis test, allowing the alternatives, X and Y, which are under comparison to have unequal variances denoted σ_x^2 and σ_y^2 respectively. With no assumption of common variance made in the formulation of the comparative decision problem, the likelihood ratio test derived herein is based upon the subsequent work of Hayre and Gittins[4].

As stated in the main text the decision maker is presented with two sources of stimuli, the neural response to which (the firing rate) is represented by two normally distributed populations X and Y from which two sequences of observations, $\mathbf{x_m} = \{x_1, \ldots x_m\}$ and $\mathbf{y_n} = \{y_1, \ldots, y_n\}$ (the firing rates), are drawn. Where, each x_i and y_j is independent and normally distributed such that $x_i \sim \mathcal{N}(\mu_x, \sigma_x^2)$ and $y_j \sim \mathcal{N}(\mu_y, \sigma_y^2)$. Stated simply, the goal of the decision maker is to determine which of the two populations has the larger mean value (μ_{High}). With two alternatives there are two possible states of nature (assuming $\mu_x \neq \mu_y$), either $\mu_x > \mu_y$ or $\mu_y > \mu_x$ which can be described by two hypotheses H_x and H_y, shown below:

$$
\begin{aligned}
H_x: &\quad \mu_x = \theta + \delta \quad \mu_y = \theta - \delta \\
H_y: &\quad \mu_x = \theta - \delta \quad \mu_y = \theta + \delta
\end{aligned}
$$

Where, θ is the average of the means of the two normally distributed variables, an unknown quantity, and 2δ is the difference in the means of the two variables, which is assumed to be known.

With the decision problem formulated as a choice between two simple hypotheses, H_x and H_y, on the basis of a fixed total number of observations, the Likelihood Ratio Test $\delta_{\mathrm{LLR}}(\mathbf{x_m}, \mathbf{y_n})$, can be formulated as follows[8]:

$$\delta_{\mathrm{LLR}}(\mathbf{x_m}, \mathbf{y_n}) = \left\{ \begin{array}{ll} H_x & \text{if } \Lambda(\mathbf{x_m}, \mathbf{y_n}) > e^{\eta} \\ H_y & \text{if } \Lambda(\mathbf{x_m}, \mathbf{y_n}) \leq e^{\eta} \end{array} \right\} \tag{1.1}$$

Where, e^{η} is the decision boundary and $\Lambda(\mathbf{x_m}, \mathbf{y_n})$, shown below, is the ratio of the likelihood of the observations $\mathbf{x_m}$ and $\mathbf{y_n}$ under the two hypotheses, H_x and H_y and e^{η} is the selected decision threshold.

$$\begin{aligned} \Lambda(\mathbf{x_m}, \mathbf{y_n}) &= \frac{\prod_{i=1}^{m} P(x_i \mid H_x) \prod_{j=1}^{n} P(y_j \mid H_x)}{\prod_{i=1}^{m} P(x_i \mid H_y) \prod_{j=1}^{n} P(y_j \mid H_y)} \\ &= \exp\left(2\delta\left(\frac{\sum x_i - m\theta}{\sigma_x^2} - \frac{\sum y_j - n\theta}{\sigma_y^2}\right)\right) \end{aligned}$$

Since the likelihood ratio stated above depends on the unknown parameter θ it is necessary to calculate a marginalised likelihood ratio which is independent of this parameter. To achieve this without making any additional assumptions it is necessary to introduce two new sets of variables $\mathbf{u_m} = \{u_2, \ldots u_m\}$ and $\mathbf{v_n} = \{v_1, \ldots v_n\}$, which are related to the x_i and y_j variables as shown below:

$$u_i = x_i - x_1, \quad i = 2, \ldots, m \qquad\qquad v_j = y_j - x_1, \quad j = 1, \ldots, n$$

Although the variables x_1, u_i and v_j are all functions of a linear combination of normally distributed random variables they are all dependent on the value x_1 and as such are no longer independent of one another. Thus, collectively the probability distrubution over the variables is given by the multivariate normal distribution[7]:

$$P(x_1, \mathbf{u_m}, \mathbf{v_n} \mid H_i) = \frac{1}{(2\pi)^{\frac{k}{2}} \mid \Sigma \mid^{\frac{1}{2}}} \exp\left(-\frac{1}{2}\left(\mathbf{x} - \mu_{H_i}\right)^T \Sigma^{-1}\left(\mathbf{x} - \mu_{H_i}\right)\right) \tag{1.2}$$

Where, $\mathbf{x}$ is a vector containing the variables x_1, $u_2 \ldots u_m$ and $v_1 \ldots v_n$, μ_{H_i} is a vector containing the means of the variables in x under hypothesis H_i and Σ is the covariance matrix of the variables, as shown in Equations 1.3 and 1.4 respectively.

$$x = \begin{bmatrix} x_1, \\ u_2, \\ \vdots \\ u_m, \\ v_1, \\ \vdots \\ v_n \end{bmatrix} \qquad\qquad \mu_{H_i} = \begin{bmatrix} \mu_{x|H_i}, \\ \mu_{u|H_i}, \\ \vdots \\ \mu_{u|H_i}, \\ \mu_{v|H_i}, \\ \vdots \\ \mu_{v|H_i} \end{bmatrix} \tag{1.3}$$

Where $\mu_{x|H_i}$, $\mu_{u|H_i}$ and $\mu_{v|H_i}$ are the mean values of the variables x, u and v under hypothesis H_i and the elements of the covariance matrix Σ are listed below and shown

in matrix form in Equation 1.4 below.

$$
\begin{aligned}
Cov\,(x_1, x_1) &= \sigma_x^2 \\
Cov\,(u_i, u_i) &= 2\sigma_x^2 \\
Cov\,(v_i, v_i) &= \sigma_x^2 + \sigma_y^2 \\
Cov\,(x_1, u_i) &= Cov\,(u_i, x_1) = -\sigma_x^2 \\
Cov\,(x_1, v_i) &= Cov\,(v_i, x_1) = -\sigma_x^2 \\
Cov\,(u_i, u_j) &= Cov\,(u_j, u_i) = \sigma_x^2 \quad \forall i \neq j \\
Cov\,(v_i, v_j) &= Cov\,(v_j, v_i) = \sigma_x^2 \quad \forall i \neq j \\
Cov\,(u_i, v_j) &= Cov\,(v_j, u_i) = \sigma_x^2 \quad \forall i \neq 0
\end{aligned}
$$

Populating the covariance matrix Σ with these terms yields:

$$
\Sigma = \begin{pmatrix}
\sigma_x^2 & -\sigma_x^2 & \cdots & \cdots & \cdots & \cdots & -\sigma_x^2 \\
-\sigma_x^2 & 2\sigma_x^2 & & & & & \\
\vdots & & \ddots & & & & \\
\vdots & & & 2\sigma_x^2 & & & \\
\vdots & & & & \sigma_x^2 + \sigma_y^2 & & \\
\vdots & & & & & \ddots & \\
-\sigma_x^2 & & & & & & \sigma_x^2 + \sigma_y^2
\end{pmatrix} \tag{1.4}
$$

To calculate the inverse of the covariance matrix (Equation 1.5) it is necessary to perform a decomposition operation first, which yields two triangular matrices, a lower triangular matrix L and an upper triangular matrix U, the product of which yields the original covariance matrix $\Sigma = LU$[9].

$$
L = \begin{pmatrix}
1 & 0 & \cdots & \cdots & \cdots & \cdots & 0 \\
-1 & \ddots & & & \mathbf{0} & & \\
\vdots & & \ddots & & & & \\
\vdots & & & \ddots & & & \\
\vdots & & & & \ddots & & \\
\vdots & & \mathbf{0} & & & \ddots & \\
-1 & & & & & & 1
\end{pmatrix}
U = \begin{pmatrix}
\sigma_x^2 & -\sigma_x^2 & \cdots & \cdots & \cdots & \cdots & -\sigma_x^2 \\
0 & \sigma_x^2 & & & & & \\
\vdots & & \ddots & & \mathbf{0} & & \\
\vdots & & & \sigma_x^2 & & & \\
\vdots & & & & \sigma_y^2 & & \\
\vdots & & \mathbf{0} & & & \ddots & \\
0 & & & & & & \sigma_y^2
\end{pmatrix}
$$

Where, the large off-diagonal zeros indicate that all remaining elements in the matrices L and U for which a value has not been specified all have value 0.

With the covariance matrix decomposed, the inverse of the matrix can be calculted in two stages. In the first step the inverse of L is calculated. Following this, the inverse of Σ is given by finding the matrix Σ^{-1} which satisfies $U\Sigma^{-1} = L^{-1}$[9].

Starting with the lower triangular matrix L, it can be seen that the matrix is an atomic-triangular matrix, a special form of triangular matrix in which all off-diagonal elements are zero with the exception of a single column which contains non-zero values. The inverse of such a matrix is found by simply replacing the non-zero off-diagonal elements with their additive inverse. Substituting the result into $U\Sigma^{-1} = L^{-1}$ yields the following result:

$$\begin{pmatrix} \sigma_x^2 & -\sigma_x^2 & \cdots & \cdots & \cdots & \cdots & -\sigma_x^2 \\ 0 & \sigma_x^2 & & & & & \\ \vdots & & \ddots & \mathbf{0} & & & \\ \vdots & & & \sigma_x^2 & & & \\ \vdots & & & & \sigma_y^2 & & \\ \vdots & \mathbf{0} & & & & \ddots & \\ 0 & & & & & & \sigma_y^2 \end{pmatrix} \Sigma^{-1} = \begin{pmatrix} 1 & 0 & \cdots & \cdots & \cdots & \cdots & 0 \\ 1 & \ddots & & & & & \\ \vdots & & \ddots & \mathbf{0} & & & \\ \vdots & & & \ddots & & & \\ \vdots & & & & \ddots & & \\ \vdots & \mathbf{0} & & & & \ddots & \\ 1 & & & & & & 1 \end{pmatrix}$$

With the exception of the first row of the matrix U, all the remaining rows contain only a single non-zero element. As such, calculation of the inverse of matrix Σ^{-1} can be performed with relative ease through a process of backwards substitution yielding:

$$\Sigma^{-1} = \begin{pmatrix} \frac{m\sigma_y^2+n\sigma_x^2}{\sigma_x^2\sigma_y^2} & \frac{1}{\sigma_x^2} & \cdots & \frac{1}{\sigma_x^2} & \frac{1}{\sigma_y^2} & \cdots & \frac{1}{\sigma_y^2} \\ \frac{1}{\sigma_x^2} & \frac{1}{\sigma_x^2} & & & & & \\ \vdots & & \ddots & & & & \\ \frac{1}{\sigma_x^2} & & & \frac{1}{\sigma_x^2} & & & \\ \frac{1}{\sigma_y^2} & & & & \frac{1}{\sigma_y^2} & & \\ \vdots & & & & & \ddots & \\ \frac{1}{\sigma_y^2} & & & & & & \frac{1}{\sigma_y^2} \end{pmatrix} \tag{1.5}$$

Substituting these terms into the exponent of the multivariate normal distribution (Equation 1.2) the following result is obtained:

$$\begin{aligned} \left(\mathbf{x} - \mu_{H_i}\right)^T \Sigma^{-1} \left(\mathbf{x} - \mu_{H_i}\right) = & \frac{\left(n\sigma_x^2 + m\sigma_y^2\right)\left(x_1 - \mu_x\right)^2}{\sigma_x^2\sigma_y^2} + \frac{2\left(x_1 - \mu_x\right)\sum_2^m\left(u_i - \mu_u\right) + \sum_2^m\left(u_i - \mu_u\right)^2}{\sigma_x^2} \\ & + \frac{2\left(x_1 - \mu_x\right)\sum_1^n\left(v_j - \mu_v\right) + \sum_1^n\left(v_j - \mu_v\right)^2}{\sigma_y^2} \end{aligned}$$

Next, setting $\mu_u = 0$, as this is true regardless of hypothesis, and rearranging to collect

terms of x_1 the following is obtained:

$$(x-\mu)^T \Sigma^{-1}(x-\mu) = \frac{2\mu_x \sum_2^m u_i + \sum_2^m u_i^2}{\sigma_x^2} + \frac{\sum_1^n v_j^2 - 2\mu_v \sum_1^n v_j + n\mu_v^2 - 2\mu_x\left(\sum_1^n v_j - n\mu_v\right)}{\sigma_y^2}$$
$$+ \frac{\mu_x^2\left(n\sigma_x^2 + m\sigma_y^2\right)}{\sigma_x^2\sigma_y^2} + \frac{x_1^2\left(n\sigma_x^2 + m\sigma_y^2\right)}{\sigma_x^2\sigma_y^2}$$
$$+ x_1\left(\frac{2\sum_2^m u_i}{\sigma_x^2} + \frac{2\left(\sum_1^n v_j - n\mu_v\right)}{\sigma_y^2} - \frac{2\mu_x\left(n\sigma_x^2 + m\sigma_y^2\right)}{\sigma_x^2\sigma_y^2}\right)$$

Following this, using the definite integral $\int_{-\infty}^{\infty} e^{-ax_1^2} e^{-2bx_1} e^c dx_1 = \sqrt{\frac{\pi}{a}} e^{\frac{b^2}{a}+c}$, the parameter x_1 can be integrated out of the likelihood function, yielding a marginal likelihood of $e^{f_{H_i}}$, the exponent of which is shown below:

$$f_{H_i} = \frac{\left(\sum_1^n v_j\right)^2 \sigma_x^4 + 2\sum_1^n v_j\left(\sum_2^m u_i + m\mu_v\right)\sigma_x^2\sigma_y^2 - \sum_1^n v_j^2\left(n\sigma_x^4 + m\sigma_x^2\sigma_y^2\right)}{2\sigma_x^2\sigma_y^2\left(n\sigma_x^2 + m\sigma_y^2\right)}$$
$$+ \frac{-\sigma_y^2\left(2n\sum_2^m u_i\mu_v\sigma_x^2 + mn\mu_v^2\sigma_x^2 - \left(\sum_2^m u_i\right)^2\sigma_y^2 + \sum_2^m u_i^2\left(n\sigma_x^2 + m\sigma_y^2\right)\right)}{2\sigma_x^2\sigma_y^2\left(n\sigma_x^2 + m\sigma_y^2\right)}$$

Where, $\mu_v = \mu_x - \mu_y$ is equal to 2δ when hypothesis H_x is the true state of nature, and -2δ when hypothesis H_y is the true state of nature. Thus, with $\mu_v = \pm 2\delta$ depending on the hypothesis, the majority of terms in f_{H_i} cancel when calculating the log-likelihood ratio $\ln\Lambda(\mathbf{x_m}, \mathbf{y_n}) = f_{H_x} - f_{H_y}$.

$$\begin{aligned}\ln\Lambda(\mathbf{x_m}, \mathbf{y_n}) &= f_{H_x} - f_{H_y} \\ &= \frac{4\delta n \sum_2^m u_i - 4\delta m \sum_1^n v_j}{n\sigma_x^2 + m\sigma_y^2}\end{aligned}$$

Returning to the original variables x_i and y_j, the log-likelihood ratio can be rewritten in the form used in the main text:

$$\begin{aligned}\ln\Lambda(\mathbf{x_m}, \mathbf{y_n}) &= \frac{4\delta n \sum_2^m [x_i - x_1] - 4\delta m \sum_1^n [y_j - x_1]}{n\sigma_x^2 + m\sigma_y^2} \\ &= \frac{4\delta n \sum_2^m x_i - 4\delta m \sum_1^n y_j - 4\delta n(m-1)x_1 + 4\delta m n x_1}{n\sigma_x^2 + m\sigma_y^2} \\ &= \frac{4\delta n}{n\sigma_x^2 + m\sigma_y^2}\sum_1^m x_i - \frac{4\delta m}{n\sigma_x^2 + m\sigma_y^2}\sum_1^n y_j \qquad (1.6)\end{aligned}$$

Finally, the decision function $\delta(\mathbf{x_m}, \mathbf{y_n})$ from Equation 1.1 can be rewritten in terms of the log-likelihood function, $\mathrm{LLR}(\mathbf{x_m}, \mathbf{y_n})$, as shown below:

$$\delta(\mathbf{x_m}, \mathbf{y_n}) = \left\{\begin{array}{ll} H_x & \text{if } \mathrm{LLR}(\mathbf{x_m}, \mathbf{y_n}) > \eta \\ H_y & \text{if } \mathrm{LLR}(\mathbf{x_m}, \mathbf{y_n}) \leq \eta \end{array}\right\} \qquad (1.7)$$

Where the decision boundary η controls for the level of bias inherent in the decision rule. With the log-likelihood ratio, a decision boundary of $\eta = 0.0$ corresponds to an unbiased decision rule, in which the decision maker accepts the hypothesis under which the observations were most likely to have occurred. With no prior knowledge of the likelihood of the two hypotheses H_x and H_y, a decision boundary of $\eta = 0$ shall be used for the ideal observer.

2 Ideal Observer - Error Rates and Optimal Sampling Allocation

In the previous section a decision function was presented for selecting which of two normally distributed populations has the greatest mean. This decision function, a log-likelihood ratio test, provides the optimal solution to the decision problem for any given decision boundary and set of observations[8].

In this section the effect the sampling strategy, q, has on the expected error rate of the ideal observer is considered. To begin, in section 2.1, the expected error rate of the ideal observer decision maker is derived as a function of the decision parameters and the sampling strategy q. Next, in section 2.2, this expected error rate is used to determine the sampling strategy which minimises the error rate.

2.1 Expected Error Rate

As outlined in Section 1, the ideal observer utilises the Log-Likelihood Ratio Test (Log-LRT) $\delta_{\text{LLR}}(\mathbf{x_m}, \mathbf{y_n})$ to determine which of the two available hypotheses H_x or H_y to accept.

$$\delta_{\text{LLR}}(\mathbf{x_m}, \mathbf{y_n}) = \left\{ \begin{array}{ll} H_x & \text{if } \text{LLR}(\mathbf{x_m}, \mathbf{y_n}) > 0 \\ H_y & \text{if } \text{LLR}(\mathbf{x_m}, \mathbf{y_n}) \leq 0 \end{array} \right\}$$

Where, $\text{LLR}(\mathbf{x_m}, \mathbf{y_n})$ is the Log-Likelihood Ratio (LLR), the value of which can be calculated from the observations $\mathbf{x_m}$ and $\mathbf{y_n}$ as follows:

$$\begin{aligned} \text{LLR}(\mathbf{x_m}, \mathbf{y_n}) &= \log \Lambda(\mathbf{x_m}, \mathbf{y_n}) \\ &= \left(\frac{4\delta}{q\sigma_y^2 + (1-q)\sigma_x^2} \right) \left((1-q) \sum x_i - q \sum y_j \right) \end{aligned} \tag{2.1}$$

Thus, at a given interrogation time T, the probability of making an error is given by the integral over the distribution of LLR between $-\infty$ and 0 if H_x is the correct hypothesis and between 0 and ∞ if H_y is the correct hypothesis. This is shown below in Equation 2.2, where ER_{H_x} and ER_{H_y} are used as short hand to denote the error

rates under each of the hypotheses for a given parameterisation of the problem:

$$\mathrm{ER}_{\mathrm{LLR}|H_x} = P(\mathrm{LLR} \leq 0 \mid H_x) \quad \mathrm{ER}_{\mathrm{LLR}|H_y} = P(\mathrm{LLR} > 0 \mid H_y) \tag{2.2}$$

With $\mathrm{LLR}(\mathbf{x_m}, \mathbf{y_n})$ given by a linear combination of the observations from the normally distributed random variables X and Y, the LLR is itself a normally distributed random variable. Where the mean of LLR, denoted $\mathbb{E}(\mathrm{LLR})$, can be calculated using the following expression:

$$\mathbb{E}(\mathrm{LLR}) = \left(\frac{4\delta q(1-q)T}{q\sigma_y^2 + (1-q)\sigma_x^2} \right) (\mu_x - \mu_y) \tag{2.3}$$

Where, $(\mu_x - \mu_y)$ is equal to 2δ when hypothesis H_x is the true state of nature, and -2δ when hypothesis H_y is the true state of nature; thus, $\mathbb{E}(\mathrm{LLR} \mid H_x) = -\mathbb{E}(\mathrm{LLR} \mid H_y)$.

Similarly, the variance of LLR, denoted Var(LLR), can be calculated using the following expression:

$$\begin{aligned}
\mathrm{Var}(\mathrm{LLR}) &= \left(\frac{4\delta q(1-q)T}{q\sigma_y^2 + (1-q)\sigma_x^2} \right)^2 \mathrm{Var}\left(\bar{x}_T - \bar{y}_T\right) \\
&= \left(\frac{4\delta q(1-q)T}{q\sigma_y^2 + (1-q)\sigma_x^2} \right)^2 \left(\frac{\sigma_x^2}{qT} + \frac{\sigma_y^2}{(1-q)T} \right) && (2.4) \\
&= \frac{16\delta^2 q(1-q)T}{q\sigma_y^2 + (1-q)\sigma_x^2} && (2.5)
\end{aligned}$$

With the expressions for the mean and variance of the Log-LLR at the decision time T, the decision functions error rates (Equation 2.3) can be stated in terms standard normal Cumulative Distrubution Function (CDF), as shown below:

$$\begin{aligned}
\mathrm{ER}_{\mathrm{LLR}|H_x} &= P(\mathrm{LLR} \leq 0 \mid H_x) & \mathrm{ER}_{\mathrm{LLR}|H_y} &= P(\mathrm{LLR} > 0 \mid H_y) \\
&= \Phi\left(\frac{-\mathbb{E}(\mathrm{LLR}|H_x)}{\sqrt{\mathrm{Var}(\mathrm{LLR})}} \right) & &= 1 - \Phi\left(\frac{-\mathbb{E}(\mathrm{LLR}|H_y)}{\sqrt{\mathrm{Var}(\mathrm{LLR})}} \right)
\end{aligned} \tag{2.6}$$

Where, the argument of the normal CDF function, $\frac{-\mathbb{E}(\mathrm{LLR}|H_i)}{\sqrt{\mathrm{Var}(\mathrm{LLR})}}$, is shown below.

$$\frac{-\mathbb{E}(\mathrm{LLR} \mid H_i)}{\sqrt{\mathrm{Var}(\mathrm{LLR})}} = -\sqrt{\frac{q(1-q)T}{(1-q)\sigma_x^2 + q\sigma_y^2}} (\mu_i - \mu_{j \neq i}) \tag{2.7}$$

2.2 Optimal Allocation of Samples

Now, with the expected error rate of the ideal observer derived, the relationship between this error rate and the chosen sampling strategy q can be considered.

To achieve this, it is first noted that $\Phi(.)$ is a monotonically increasing function of its argument and, as such, the minima and maxima of the function coincides with the minimum and maximum extremum of the argument, respectively. Considering the two error rates, ER_{H_x} and ER_{H_y} individually, it can be seen that ER_{H_x} is minimised when $\frac{-\mathbb{E}(\mathrm{LLR}|H_x)}{\sqrt{\mathrm{Var}(\mathrm{LLR})}}$ is minimised and ER_{H_x} is minimised when $\frac{-\mathbb{E}(\mathrm{LLR}|H_y)}{\sqrt{\mathrm{Var}(\mathrm{LLR})}}$ is maximised.

However, since $\frac{-\mathbb{E}(\mathrm{LLR}|H_x)}{\sqrt{\mathrm{Var}(\mathrm{LLR})}} = \frac{\mathbb{E}(\mathrm{LLR}|H_y)}{\sqrt{\mathrm{Var}(\mathrm{LLR})}}$, the minimum of $\frac{-\mathbb{E}(\mathrm{LLR}|H_x)}{\sqrt{\mathrm{Var}(\mathrm{LLR})}}$ and the maximum of $\frac{\mathbb{E}(\mathrm{LLR}|H_y)}{\sqrt{\mathrm{Var}(\mathrm{LLR})}}$ coincide. Thus, any sampling strategy that minimises the error rate under one hypothesis also minimises the error rate under the other hypothesis.

Taking the derivative of $\frac{-\mathbb{E}(\mathrm{LLR}|H_x)}{\sqrt{\mathrm{Var}(\mathrm{LLR})}}$, the result of which is shown below, the optimal sampling strategy q^* can be found as follows: If stationary points in the interval $[0,1]$ are found and one or more of them is a minimum extremum then by comparing the error rate at each point the optimal strategy q^* can be found. However, if no minimum extremum exists in the range $[0,1]$, then the optimal strategy shall lie at either $q^*=0$ or $q^*=1$, with further analysis required.

$$\frac{d}{dq}\frac{-\mathbb{E}(\mathrm{LLR})}{\sqrt{\mathrm{Var}(\mathrm{LLR})}} = -\frac{(1-q)qT^2\delta\left((1-q)^2\sigma_x^2 - q^2\sigma_y^2\right)}{\left(q(1-q)^2T\sigma_x^2 + q^2(1-q)T\sigma_y^2\right)^{3/2}} \tag{2.8}$$

Now, before solving for stationary points where $\frac{d}{dq}\frac{-\mathbb{E}(\mathrm{LLR})}{\sqrt{\mathrm{Var}(\mathrm{LLR})}} = 0$ it is first noted that at certain points ($q=0$ and $q=1$) in the interval $[0,1]$ the derivative is undefined and a vertical tangent exists.

With that noted the derivative can be simplified by the multiplying out the denominator of the two terms on the Right-Hand Side (RHS) of Equation 2.8:

$$0 = -(1-q)qT^2\delta\left((1-q)^2\sigma_x^2 - q^2\sigma_y^2\right) \tag{2.9}$$

From the equation above it appears the DV has critical points at the following q values: $q=0$, $q=1$, $q=\frac{\sigma_x}{\sigma_x+\sigma_y}$ and $q=\frac{\sigma_x}{\sigma_x-\sigma_y}$. To determine which, if any, of the points correspond to valid sampling strategies and a minimum extremum of the DV requires further analysis. Starting with the points $q=0$ and $q=1$ mentioned earlier, it can be seen by substituting these values back into Equation 2.8 that these points are not in fact stationary points but correspond to vertical tangents where the derivative is undefined with $\frac{d}{dq}\frac{-\mathbb{E}(\mathrm{LLR})}{\sqrt{\mathrm{Var}(\mathrm{LLR})}} = \infty$.

Next, from inspection of the point $q=\frac{\sigma_x}{\sigma_x-\sigma_y}$ it can be seen that the point lies outside of the interval of valid sampling strategies $[0,1]$, leaving only the point $q=\frac{\sigma_x}{\sigma_x+\sigma_y}$, which does correspond to a valid sampling strategy for any σ_x and σ_y.

To check if the point $q=\frac{\sigma_x}{\sigma_x+\sigma_y}$ is a maxima, minima or a point of inflection requires the evaluation of the second derivative at $q=\frac{\sigma_x}{\sigma_x+\sigma_y}$, with $\frac{d^2}{dq^2}\frac{-\mathbb{E}(\mathrm{LLR})}{\sqrt{\mathrm{Var}(\mathrm{LLR})}} > 0$ indicating

a minima, $\frac{d^2}{dq^2}\frac{-\mathbb{E}(\text{LLR})}{\sqrt{\text{Var}(\text{LLR})}} < 0$ indicating a maxima and $\frac{d^2}{dq^2}\frac{-\mathbb{E}(\text{LLR})}{\sqrt{\text{Var}(\text{LLR})}} = 0$ requiring further analysis. Calculating the second derivative of $\frac{-\mathbb{E}(\text{LLR})}{\sqrt{\text{Var}(\text{LLR})}}$ the following result is obtained:

$$\frac{d^2}{dq^2}\frac{-\mathbb{E}(\text{LLR})}{\sqrt{\text{Var}(\text{LLR})}} = -\frac{(-1+q)qT^3\delta\left((-1+q)^4\sigma_x^4 - 2q\left(-2+3q-2q^2+q^3\right)\sigma_x^2\sigma_y^2 + q^4\sigma_y^4\right)}{2\left((-1+q)qT\left((-1+q)\sigma_x^2 - q\sigma_y^2\right)\right)^{5/2}}$$

Substituting $q = \frac{\sigma_x}{\sigma_x+\sigma_y}$ into this result and simplying yields the following result:

$$\frac{d^2}{dq^2}\frac{-\mathbb{E}(\text{LLR})}{\sqrt{\text{Var}(\text{LLR})}} \mid q = \frac{\sigma_x}{\sigma_x+\sigma_y} = \frac{2T\delta}{\sqrt{\frac{T\sigma_x^2\sigma_y^2}{(\sigma_x+\sigma_y)^2}}} \tag{2.10}$$

Inspection of this result reveals that the second derivative of $\frac{-\mathbb{E}(\text{LLR})}{\sqrt{\text{Var}(\text{LLR})}}$ at the point $q = \frac{\sigma_x}{\sigma_x+\sigma_y}$ is always positive. Thus, the point $q = \frac{\sigma_x}{\sigma_x+\sigma_y}$ corresponds to a minimum extremum and the optimal sampling strategy under either hypothesis is $q^* = \frac{\sigma_x}{\sigma_x+\sigma_y}$.

3 Ideal Observer Model - Switching Costs

In Section 2, when the solution to the decision problem as seen by the ideal observer was derived, it was assumed that the full decision time T was spent observing one or other of the two available sources of stimuli. A consequence of this assumption is that the decision maker must have the ability to instantaneously switch sampling between the two sources of stimuli.

In decisions involving visual stimuli, eye movements form an essential part of how sensory and cognitive attention is controlled during information gathering. For the participants tackling the visual discrimination task outlined in the main text, completion of these eye movements is clearly not instantaneous. Moreover, peri-saccadic suppression is likely to prolong the period in which vision is attenuated beyond the duration of the movement itself[10]. To understand the effect these non-instantaneous switches in fixation have on the behaviour of the decision maker a switching cost, in the form of a loss of sampling time, is introduced into the ideal observer model. This section investigates the effect the switching cost has on the expected behaviour and performance of the ideal observer decision maker.

If we begin by denoting the loss in sampling time by T_c and the number of switches by n_s then we can reformulate the log likelihood equation, LLR_T, as shown below.

$$\text{LLR}_T = \left(\frac{4\delta(q-q^2)(T-n_sT_c)}{q\sigma_y^2+(1-q)\sigma_x^2}\right)(\bar{x}_T - \bar{y}_T) \tag{3.1}$$

Where, q represents the portion of the utilised sampling time $T - n_s T_c$ spent drawning observations from alternative X with the total number of discrete samples drawn by the ideal observer from alternative X given by $m = q(T - n_s T_c)$ and the total number of samples drawn from alternative Y is given by $n = (1 - q)(T - n_s T_c)$.

As before (Equation 2.6), the expected error rate for the modified ideal observer can be calculated from the standard normal's CDF, as shown below.

$$\begin{aligned} \mathrm{ER}_{\mathrm{LLR}|H_x} &= P(\mathrm{LLR} \leq 0 \mid H_x) & \mathrm{ER}_{\mathrm{LLR}|H_y} &= P(\mathrm{LLR} > 0 \mid H_y) \\ &= \Phi\left(\frac{-\mathbb{E}(\mathrm{LLR}|H_x)}{\sqrt{\mathrm{Var}(\mathrm{LLR})}}\right) & &= 1 - \Phi\left(\frac{-\mathbb{E}(\mathrm{LLR}|H_y)}{\sqrt{\mathrm{Var}(\mathrm{LLR})}}\right) \end{aligned} \tag{3.2}$$

Where the argument of the $\Phi(.)$ is given as follows:

$$\frac{\eta - \mathbb{E}(\mathrm{LLR} \mid H_i)}{\sqrt{\mathrm{Var}(\mathrm{LLR})}} = -\frac{q(1-q)(T - n_s T_c)\,(\mu_x - \mu_y)}{\sqrt{\left(q(1-q)^2(T - n_s T_c)\sigma_x^2 + q^2(1-q)(T - n_s T_c)\sigma_y^2\right)}} \tag{3.3}$$

As noted previously in Section 2.2, the expected error rate is minimised under H_x when the argument of $\Phi(.)$ has minimum value and is minimised under H_y when the argument of $\Phi(.)$ has maximum value. Inspection of Equation 3.3 shows that increasing the value of n_s leads to a increase in the expected error rate achieved under both hypothesis H_x and H_y. Next, we note an additional property of introducing switching costs, which is that for values of $n_s = 0$ the sampling proportion q must be equal to either 0 or 1, as a switch must occur in order for samples to be drawn from both of the alternatives. Thus, from Equations 3.2 and 3.3, we can see that at $n_s = 0$ $\frac{\eta - \mathbb{E}(\mathrm{LLR}|H_i)}{\sqrt{\mathrm{Var}(\mathrm{LLR})}} = 0$ and an expected error rate of 50% is achieved. Further inspection shows that whenever $T > T_c$, setting $n_s = 1$ and having a $0 < q < 1$ leads to a reduction in error rate compared to that achieved when $n_s = 0$.

Next, to determine the optimal sampling strategy to be used for a given problem parameterisation, we note that the introduction of a switching penalty has merely resulted in a reduction the amount of effective sampling time available. Since the optimal sampling strategy, $q = \frac{\sigma_x}{\sigma_x + \sigma_y}$, derived in the previous section is independent of sampling time and depends only on the values of σ_x and σ_y we can conclude that the introduction of a switching penalty has no effect on how the available sampling time should be apportioned between the variables.

From which we can conclude that, in the situation when values of σ_x and σ_y are known and the switching cost satisfies the inequality $T_c < T$, the expected error rate is minimised by selecting $n_s = 1$ and $q = \frac{\sigma_x}{\sigma_x + \sigma_y}$.

Relating this optimal one-switch strategy back to the number of samples, m and n, drawn from each of the two variables X and Y we find that $m = q(T - T_c)$, $n = (1 - q)(T - T_c)$ and $T = m + n + T_c$.

4 Ideal Observer - Unknown Variance

In the previous section it was noted that the ideal observer model made an implicit assumption that the decision maker is capable of instantaneously switching fixation between the two available sources of stimuli. As noted, this assumption is unrealistic for the human participants solving visual discrimination tasks. Thus, a switching cost, in the form of a loss of useable sampling time for each switch effected, was incorporated into the ideal observer model.

In this section another assumption made about the decision makers is considered, namely that of known stimuli noise. Recall that the optimal strategy $q = \frac{\sigma_x}{\sigma_x+\sigma_y}$ depends on relative noise levels of the stimuli. From a theoretical standpoint, optimal solutions do not exist for decisions requiring inferences about the means of two normal populations when the variances of each population are not necessarily equal and the variances themselves (or their ratio) are unknown[6]. Therefore, in order to simplify the problem we consider an ideal case in which there are two variances σ^2_{low} and σ^2_{high} (as in the visual discrimination task described in the main text) and the decision making agent is an ideal observer, which after a brief sampling period, is able to classify the information source as low or high noise. Furthermore, it is assumed that the two variances are equally likely for each of the alternatives such that the 4 possible combinations with which the variances of the two sources can be drawn from $\{\sigma^2_{\text{low}}, \sigma^2_{\text{high}}\}$ are all equally likely.

Under such a formulation, the challenge of the agent then becomes how to apportion the available sampling time between the two alternatives, given that the variance of the second alternative will be unknown at the time the switch is made. In order to further simplify the task we shall begin by assuming that the agent makes only a single switch between the two alternatives; later, in Section 4.2, we will consider under what conditions the agent's performance would be best served by making multiple switches.

4.1 Single Switch

Given this formulisation of the unknown variance case and assuming, without loss of generality that X is initially fixated upon such that σ_x is known σ_y unknown, the expected error rate of the decision maker can be written as follows:

$$\begin{aligned} \text{ER}(q,T) = {} & P_Y(\sigma^2_{\text{low}})\text{ER}(\sigma^2_x, \sigma^2_y = \sigma^2_{\text{low}}, q, T, \delta) \\ & + P_Y(\sigma^2_{\text{high}})\text{ER}(\sigma^2_x, \sigma^2_y = \sigma^2_{\text{high}}, q, T, \delta) \end{aligned} \tag{4.1}$$

Where, $P_Y(\sigma^2_{\text{low}})$ and $P_Y(\sigma^2_{\text{high}})$ are the prior probabilities that $\sigma^2_y = \sigma^2_{\text{low}}$ and $\sigma^2_y = \sigma^2_{\text{high}}$ respectively.

With the expected error rate dependent on the value of σ_x, there are two distinct error

rates, which shall be denoted $\text{ER}(q,T)_{|\sigma_x=\sigma_{\text{low}}}$ and $\text{ER}(q,T)_{|\sigma_x=\sigma_{\text{high}}}$. Considering the case $\sigma_x^2=\sigma_{\text{low}}^2$ first, the value of q which yields the smallest expected error rate can be found by taking the derivative of Equation 4.1, shown below, and solving for stationary points.

$$\frac{\partial}{\partial q}ER(q,T)_{|\sigma_x=\sigma_{\text{low}}^2} = \frac{e^{\frac{2(q-1)qT\delta^2}{\sigma_{\text{low}}^2}}(2q-1)\sqrt{-\frac{(q-1)qT\delta^2}{\sigma_{\text{low}}^2}}}{2\sqrt{2\pi}(q-1)q} + \frac{e^{-\frac{2(q-1)qT\delta^2}{(q-1)\sigma_{\text{low}}^2-q\sigma_{\text{high}}^2}}T\delta^2\left((q-1)^2\sigma_{\text{low}}^2-q^2\sigma_{\text{high}}^2\right)}{2\sqrt{2\pi}\sqrt{\frac{(q-1)qT\delta^2}{(q-1)\sigma_{\text{low}}^2-q\sigma_{\text{high}}^2}}\left((q-1)\sigma_{\text{low}}^2-q\sigma_{\text{high}}^2\right)^2}$$

Unfortunately, it is not possible to determine analytically the values of q which correspond to the stationary points of the function. However, although we cannot determine the exact value of q which corresponds to the minimum extremum of the error function we can be sure that it's value lies between the optimal value for the cases $\sigma_y=\sigma_{\text{low}}$ and $\sigma_y=\sigma_{\text{high}}$, as shown below.

$$\frac{\sigma_{\text{low}}}{\sigma_{\text{low}}+\sigma_{\text{high}}} < q^* < \frac{\sigma_{\text{low}}}{\sigma_{\text{low}}+\sigma_{\text{low}}} \tag{4.2}$$

This inequality follows from the analysis of the ideal observers error rate in Section 2, where it was shown that the error rate for the known variance case has a single turning point within the acceptable range of sampling strategies ($0 \le q \le 1$). With that point ($q=\frac{\sigma_x}{\sigma_x+\sigma_y}$) corresponding to a minimum of the error rate, deviations in sampling strategy from $q=\frac{\sigma_x}{\sigma_x+\sigma_y}$ within the range $0\le q\le 1$ result in an increase in error rate with larger deviations yielding a larger increases in error rate.

Thus, as q approaches the lower bound of the inequality from below ($q<\frac{\sigma_{\text{low}}}{\sigma_{\text{low}}+\sigma_{\text{high}}}$), the argument of the normal CDF function ($\frac{-\mathbb{E}(\text{LLR}|H_i)}{\sqrt{\text{Var(LLR)}}}$) and thus the error rate for both $\sigma_y^2=\sigma_{\text{low}}^2$ and $\sigma_y^2=\sigma_{\text{high}}^2$, will be decreasing. Conversely, as q moves past the upper bound ($q>\frac{\sigma_{\text{low}}}{\sigma_{\text{low}}+\sigma_{\text{low}}}$) the argument and error rate for both $\sigma_y^2=\sigma_{\text{low}}^2$ and $\sigma_y^2=\sigma_{\text{high}}^2$, will be increasing. As the value of q is varied from the lower bound to the upper bound the argument and error rate for increase for $\sigma_y^2=\sigma_{\text{low}}^2$ and decrease for $\sigma_y^2=\sigma_{\text{low}}^2$. Thus, since the expected error rate for the unknown variance case is a weighted average of the error rates for the two known variance cases, there exists a point between these two bounds at which the expected overall error rate is minimised.

Similarly to the above, when $\sigma_x^2=\sigma_{\text{high}}$ the region containing the optimal strategy q^* is bounded as follows:

$$\frac{\sigma_{\text{high}}}{\sigma_{\text{high}}+\sigma_{\text{high}}} < q^* < \frac{\sigma_{\text{high}}}{\sigma_{\text{high}}+\sigma_{\text{low}}} \tag{4.3}$$

In Figure 4.1(b) the two regions containing the optimal sampling strategy for the conditions of $\sigma_x^2 = \sigma_{\text{low}}$ and $\sigma_x^2 = \sigma_{\text{high}}$ are illustrated along with the optimal strategies for the four known variances cases (Figure 4.1(a)) for comparison.

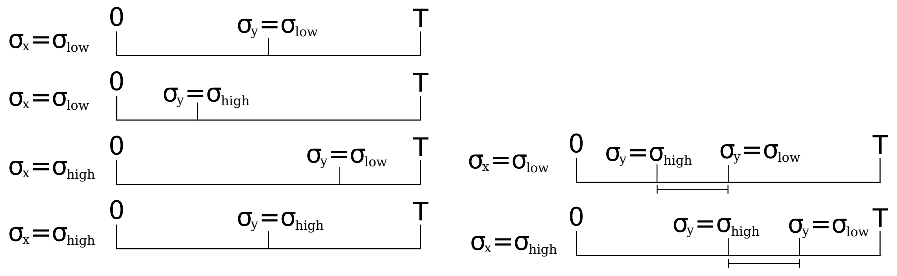

(a) $\sigma_x = \sigma_{\text{low}}, \sigma_y = \sigma_{\text{low}}$ (b) $\sigma_x = \sigma_{\text{High}}, \sigma_y = \sigma_{\text{low}}$

Figure 4.1: Division of sampling time for varying combinations of high and low noise stimuli for instantaneous switching decision problems. In Panel (a) the timelines show the division of sampling time between the sources of stimuli for the four possible combinations of high and low noise stimuli with the noise of both sources known a priori. In each timeline we show the simplest single switch strategy, the timing of the switch is indicated by the vertical line, with alternative X observed from time 0 to the switching point and alternative Y observed thereafter until the trial ends at time T. In Panel (b) the timelines show the range of times in which the optimal first switching point lies when the noise level of stimulus Y is unknown. Unlike the known variance case, the exact location of the optimal first switching point depends on not only the two possible noise levels but also depends on the total sampling time available and 2δ, the difference in the response to the stimuli.

From inspection of Figure 4.1 and the bounds given in Equations 4.2 and 4.3 it can be seen that the regions of the sampling space containing the optimal sampling strategy under the two conditions $\sigma_x^2 = \sigma_{\text{low}}$ and $\sigma_x^2 = \sigma_{\text{high}}$ do not overlap. Thus, for $\sigma_{\text{low}} \neq \sigma_{\text{high}}$, the optimal sampling strategy cannot be determined until X has been sampled and the value of σ_x is known.

4.2 Multiple Switches

In the previous section the effect unknown variances has on the optimal sampling strategy when the decision maker is restricted to making only a single switch between the sources of stimuli was investigated. Under such conditions it was shown that, whilst the optimal strategy itself cannot be found analytically, it does lie between the bounds

outlined below:

$$\begin{aligned}\frac{\sigma_{\text{low}}}{\sigma_{\text{low}}+\sigma_{\text{high}}} \leq q^* \leq \frac{\sigma_{\text{low}}}{\sigma_{\text{low}}+\sigma_{\text{low}}} \quad &\text{if } \sigma_x = \sigma_{\text{low}} \\ \frac{\sigma_{\text{high}}}{\sigma_{\text{high}}+\sigma_{\text{high}}} \leq q^* \leq \frac{\sigma_{\text{high}}}{\sigma_{\text{high}}+\sigma_{\text{low}}} \quad &\text{if } \sigma_x = \sigma_{\text{high}}\end{aligned} \tag{4.4}$$

In this section we consider under what conditions the decision maker should choose to make additional switches in fixation given that the chosen switching time lies between the bounds identified above. In addition, the influence the switching cost has on the switching frequency is considered.

Assuming that the initial switch has already been effected and the variance of both alternatives are known to the decision maker, a second switch will be beneficial if the expected error rate with the second switch is less than without. Denoting the useable sampling time and sampling strategy by T_1 and q_1 for the single switch case and by T_2 and q_2 for the two switch strategy this can be written as follows:

$$\text{ER}(\sigma_x^2, \sigma_y^2, q_2, T_2, \delta) < \text{ER}(\sigma_x^2, \sigma_y^2, q_1, T_1, \delta) \tag{4.5}$$

In addition to the previous assumptions that have been stated it can also be assumed that $T_2 \leq T_1$ with equality between the sampling times when there is no switching cost ($T_c = 0$). Furthermore, since switching back to alternative X can only increase the number of samples drawn from X, it holds that $q_2T_2 > q_1T_1$ (number of samples from X given by $m = qT$) thus, since $T_2 \leq T_1$, q_2 must be strictly larger than q_1.

Now, by considering the four possible combinations of variance individually, Equation 4.5, and the benefit of an additional switch, can be evaluated. Starting with $\sigma_x = \sigma_{\text{low}}$ and $\sigma_y = \sigma_{\text{high}}$ we know, from Section 3, that the optimal sampling strategy $q^* = \frac{\sigma_{\text{low}}}{\sigma_{\text{low}}+\sigma_{\text{high}}}$. As noted in the previous section, deviations in sampling strategy from $q^* = \frac{\sigma_x}{\sigma_x+\sigma_y}$ within the range $0 \leq q \leq 1$ result in an increase in error rate with a larger deviations yielding a larger error rate increase. Thus, since it has been assumed that the switching time q_1 lies within the bounds in Equation 4.4 and since $q_1 < q_2$, even if $T_c = 0$, additional switches can only serve to increase the error rate. Likewise, with $\sigma_x = \sigma_{\text{high}}$ and $\sigma_y = \sigma_{\text{high}}$ the optimal sampling strategy again corresponds to the lower bound with $q^* = \frac{\sigma_{\text{low}}}{\sigma_{\text{low}}+\sigma_{\text{high}}}$ and additional switches can only serve to increase the error rate.

Conversely, when presented with the combinations of noise of $\sigma_x = \sigma_{\text{low}}$ and $\sigma_y = \sigma_{\text{low}}$ or $\sigma_x = \sigma_{\text{high}}$ and $\sigma_y = \sigma_{\text{low}}$ if the first switch strategy lies below the upper bound outlined in Equation 4.4 then an additional switch (increase portion of time allocated to alternative X) could serve to improve the performance of the decision maker. Determining exactly when a second switch is beneficial requires further analysis of the inequality in Equation 4.5. Before considering two remaining variance combinations individually it is first noted that, since the error rates, under both H_x and H_y, are monotonically increasing functions of $\frac{-\mathbb{E}(\text{LLR}|H_i)}{\sqrt{\text{Var}(\text{LLR})}}$, an equivalent inequality for deter-

mining the efficiacy of an additional switch is given as follows:

$$-\frac{2(1-q_2)q_2T_2(\mu_x-\mu_y)}{\sqrt{(1-q_2)^2q_2T_2\sigma_x^2+(1-q_2)q_2^2T_2\sigma_y^2}} < -\frac{2(1-q_1)q_1T_1(\mu_x-\mu_y)}{\sqrt{(1-q_1)^2q_1T_1\sigma_x^2+(1-q_1)q_1^2T_1\sigma_y^2}} \tag{4.6}$$

Assuming that it is beneficial to the decision maker to make a second switch then, from Section 2, it is clear that the decision maker will minimise their error rate with total sampling time T_2 by allocating samples such that $q_2 = \frac{\sigma_x}{\sigma_x+\sigma_y}$. Given this assumption and the simplified inequality above, the conditions under which an additional switch is beneficial can be considered for the two remaining combinations of variance.

Starting with the case when $\sigma_x = \sigma_{\text{low}}$ and $\sigma_y = \sigma_{\text{low}}$ and substituting in the optimal 2-switch sampling allocation of $q_2 = \frac{\sigma_{\text{low}}}{\sigma_{\text{low}}+\sigma_{\text{high}}}$ the inequality simplifies as follows:

$$\begin{aligned}
-\frac{T_2(\mu_x-\mu_y)}{\sqrt{T_2\sigma_L^2}} &< -\frac{2(1-q_1)q_1T_1(\mu_x-\mu_y)}{\sqrt{(1-q_1)^2q_1T_1\sigma_L^2+(1-q_1)q_1^2T_1\sigma_L^2}}\\
\frac{\sqrt{T_2}}{\sigma_{\text{low}}} &> \frac{2(1-q_1)q_1\sqrt{T_1}}{\sqrt{(1-q_1)^2q_1\sigma_{\text{low}}^2+(1-q_1)q_1^2\sigma_{\text{low}}^2}}\\
T_2 &> 4T_1(1-q_1)q_1
\end{aligned}$$

Since $4(1-q_1)q_1$ lies in the interval $[0,1]$ it can be seen that with no switching cost ($T_2 = T_1$) the inequality is satisfied and an additional switch is always benefical and as the switching cost grows the inequality will eventually fail to be satisfied and a further switch becomes detrimental to the error rate.

Finally, considering the case with $\sigma_x = \sigma_{\text{high}}$ and $\sigma_y = \sigma_{\text{low}}$ and substituting in the optimal 2-switch sampling allocation of $q_2 = \frac{\sigma_{\text{high}}}{\sigma_{\text{high}}+\sigma_{\text{low}}}$ the inequality simplifies as follows:

$$\begin{aligned}
-\frac{2(\mu_x-\mu_y)\sqrt{\frac{T_2\sigma_{\text{high}}^2\sigma_{\text{low}}^2}{(\sigma_{\text{high}}+\sigma_{\text{low}})^2}}}{\sigma_{\text{high}}\sigma_{\text{low}}} &< -\frac{2(1-q_1)q_1T_1(\mu_x-\mu_y)}{\sqrt{(1-q_1)^2q_1T_1\sigma_{\text{high}}^2+(1-q_1)q_1^2T_1\sigma_{\text{low}}^2}}\\
\sqrt{\frac{T_2}{(\sigma_{\text{high}}+\sigma_{\text{low}})^2}} &> \sqrt{\frac{(1-q_1)q_1T_1}{(1-q_1)\sigma_{\text{high}}^2+q_1\sigma_{\text{low}}^2}}\\
T_2 &> \frac{\left(q_1\sigma_{\text{high}}^2-q_1^2\sigma_{\text{high}}^2+2q_1\sigma_{\text{high}}\sigma_{\text{low}}-2q_1^2\sigma_{\text{high}}\sigma_{\text{low}}\right)T_1}{\sigma_{\text{high}}^2-q_1\sigma_H^2+q_1\sigma_{\text{low}}^2}
\end{aligned} \tag{4.7}$$

In summary, if the second sampled alternative is the more noisy one, i.e. $\sigma_y = \sigma_{high}$, the accuracy is maximized by a single switch, but if $\sigma_y = \sigma_{low}$, an additional switch may improve accuracy under certain conditions.

5 DV Decision Maker - Error Rates and Optimal Sampling Allocation

In the main text it was shown how the log-likelihood ratio used in the ideal observer model (derived in Section 1) could be simplified to yield a decision variable (DV). Importantly, this simplified model was shown to have equivalent performance to the ideal observer model when particular sampling strategies were utilised.

In this section the expected error rate for the DV decision maker and the relationship between this error rate and the sampling strategy used is considered. To begin, in section 5.1 the expected error rate for the DV decision maker is derived. Next, in section 5.2 this error rate is related to that of the ideal observer. Finally, in section 5.3 the effect deviations from the optimal sampling strategy has on the performance of the decision maker is considered.

5.1 Expected Error Rate

In this section an expression is derived giving the expected error rate of the DV decision maker in terms of the decision problem parameters and the free parameter q which dictates the sampling strategy. As outlined in the main text, the DV decision maker utilises the decision function $\delta_{\mathrm{DV}}(\mathbf{x_m}, \mathbf{y_n})$ to determine which of the two available hypotheses H_x or H_y to accept:

$$\delta_{\mathrm{DV}}(\mathbf{x_m}, \mathbf{y_n}) = \left\{ \begin{array}{ll} H_x & \text{if } \mathrm{DV}(\mathbf{x_m}, \mathbf{y_n}) > 0 \\ H_y & \text{if } \mathrm{DV}(\mathbf{x_m}, \mathbf{y_n}) \leq 0 \end{array} \right\}$$

Throughout this section $\mathrm{DV}(\mathbf{x_m}, \mathbf{y_n})$ and DV shall be used interchangeably to refer to the value of the decision variable having made the observations $\mathbf{x_m}$ and $\mathbf{y_n}$; which can be calculated as follows:

$$\begin{aligned} \mathrm{DV}(\mathbf{x_m}, \mathbf{y_n}) &= \sum x_i - w \sum y_j \\ &= qT\bar{x} - w(1-q)T\bar{y} \end{aligned} \tag{5.1}$$

Where, $\bar{x}$ and $\bar{x}$ are the mean values of the observations made from sources X and Y respectively and $w = \frac{\tilde{q}}{1-\tilde{q}}$, with $\tilde{q}$ representing the assumed or planned sampling strategy.

Thus, as with the optimal observer error rate, at a given interrogation time T, the probability of making an error is given by the integral over the distribution of DV between $-\infty$ and 0 if H_x is the correct hypothesis and between 0 and ∞ if H_y is the correct hypothesis. This is shown below in Equation 5.2, where ER_{H_x} and ER_{H_y} are used as short hand to denote the error rates under each of the hypotheses for a given

parameterisation of the problem:

$$\mathrm{ER}_{\mathrm{DV}|H_x} = P(\mathrm{DV} \leq 0 \mid H_x) \quad \mathrm{ER}_{\mathrm{DV}|H_y} = P(\mathrm{DV} > 0 \mid H_y) \tag{5.2}$$

Again, as with the optimal observer, the decision variable, like the LLR, is given by a linear combination of the observations from the normally distributed random variables X and Y. Thus the decision variable is itself a normally distributed random variable. Denoting the expected value of DV by $\mathbb{E}[\mathrm{DV}]$ and the variance by Var(DV), they can each be calculated from the decision problem parameters as follows:

$$\begin{aligned}\mathbb{E}[\mathrm{DV}] &= qT\mu_x - w(1-q)T\mu_y \\ \mathrm{Var}(\mathrm{DV}) &= qT\sigma_x^2 + w^2(1-q)T\sigma_y^2\end{aligned}$$

With the expressions for the mean and variance of the DV at the decision time T formulated, the DV error rates (Equation 5.2) can be stated in terms of the standard normal CDF, as shown below:

$$\begin{aligned}\mathrm{ER}_{H_x} &= P(\mathrm{DV} \leq 0 \mid H_x) & \mathrm{ER}_{H_y} &= P(\mathrm{DV} > 0 \mid H_y) \\ &= \Phi\left(\frac{-\mathbb{E}(\mathrm{DV}|H_x)}{\sqrt{\mathrm{Var}(\mathrm{DV})}}\right) & &= 1 - \Phi\left(\frac{-\mathbb{E}(\mathrm{DV}|H_y)}{\sqrt{\mathrm{Var}(\mathrm{DV})}}\right)\end{aligned} \tag{5.3}$$

Where, the argument of the normal CDF function, $\frac{-\mathbb{E}(\mathrm{DV}|H_i)}{\sqrt{\mathrm{Var}(Z_T)}}$, is shown below:

$$\frac{-\mathbb{E}(\mathrm{DV})}{\sqrt{\mathrm{Var}(\mathrm{DV})}} = -\frac{qT\mu_x - w(1-q)T\mu_y}{\sqrt{qT\sigma_x^2 + w^2(1-q)T\sigma_y^2}} \tag{5.4}$$

In the next section this result and the equivalent result for the ideal observer (Equation 2.7) shall be compared to establish how the error rate of the DV decision maker relates to that of the optimal ideal observer decision maker and to determine how the DV decision maker should allocate their samples to minimise the expected error rate.

5.2 Relationship to Ideal Observer

In the previous section an expression was derived giving the expected error rate of the DV decision maker in terms of the decision problem parameters and the free parameter q which dictates the sampling strategy. In this section the DV error rate expression along with the stages used to derive the DV decision maker from the ideal observer decision maker are used to relate the performance of the decision makers and determine the optimal sampling allocation the DV decision maker should utilise.

In the main text it was shown that when the DV decision maker implements the planned sampling strategy of $\tilde{q}$ ($q = \tilde{q}$) the ideal observer and DV decision maker behave identically. With the error rates of the ideal observer (Equation 2.6) and DV decision maker (Equation 5.3) both given by the standard normal CDF ($\Phi(.)$)

this relationship can be seen by comparing the values of $\frac{-\mathbb{E}(\text{LLR})}{\sqrt{\text{Var(LLR)}}}$ and $\frac{-\mathbb{E}(\text{DV})}{\sqrt{\text{Var(DV)}}}$. Inspection of these functions, which are shown below and examples of which are plotted in Figure 5.1, reveals that as expected the error rates intersect when the DV decision maker implements the planned sampling strategy of $\tilde{q}$

$$\begin{aligned}\frac{-\mathbb{E}(\text{DV})}{\sqrt{\text{Var(DV)}}} &= -\frac{qT\mu_x - \frac{\tilde{q}}{1-\tilde{q}}(1-q)T\mu_y}{\sqrt{qT\sigma_x^2 + \left(\frac{\tilde{q}}{1-\tilde{q}}\right)^2(1-q)T\sigma_y^2}} \\ &= -\frac{q(1-\tilde{q})T\mu_x - \tilde{q}(1-q)T\mu_y}{\sqrt{q(1-\tilde{q})^2T\sigma_x^2 + \tilde{q}^2(1-q)T\sigma_y^2}}\end{aligned}$$

$$\frac{-\mathbb{E}(\text{LLR})}{\sqrt{\text{Var(LLR)}}} = -\frac{q(1-q)T\mu_x - q(1-q)T\mu_y)}{\sqrt{q(1-q)^2T\sigma_x^2 + q^2(1-q)T\sigma_y^2}}$$

As discussed previously, the log-likelihood ratio used by the ideal observer is the optimal decision strategy for two-alternative decisions made with a specified decision threshold (η) on the basis of a fixed number of observations[8]. Given the optimality of the ideal observer and the relationship between the error rates of the ideal observer and DV decision maker identified above it can be seen that DV decision maker's performance is maximised when implementing the planned sampling strategy $\tilde{q}$. Furthermore, since $q^* = \frac{\sigma_x}{\sigma_x+\sigma_y}$ yields the optimal performance for the ideal observer, planning and implementing a sampling strategy of $q = \tilde{q} = q^*$, will also yield the optimal error rates for the DV decision maker as well.

5.3 Deviation from Planned Strategy

In the previous section it was shown that the DV decision maker and ideal observer are equivalent whenever the planned sampling strategy, $\tilde{q}$, coincides with the actual sampling strategy q. Furthermore, since a sampling strategy of $q^* = \frac{\sigma_x}{\sigma_x+\sigma_y}$ was shown to minimise the error rate for the ideal observer and since the ideal observer provides the optimal solution to the decision problem [8] this strategy also minimises the error rate for the DV decision maker when $\tilde{q} = q = \frac{\sigma_x}{\sigma_x+\sigma_y}$. In this section the performance of the DV decision maker when deviating from the planned sampling strategy is considered.

As shown in Section 5.1 the DV decision maker's error rate, like the ideal observer's, is a function of standard normals cumulative probability distribution ($\Phi(.)$):

$$\begin{aligned}\text{ER}_{H_x} &= P(\text{DV} \leq 0 \mid H_x) & \text{ER}_{H_y} &= P(\text{DV} > 0 \mid H_y) \\ &= \Phi\left(\frac{-\mathbb{E}(\text{DV}|H_x)}{\sqrt{\text{Var(DV)}}}\right) & &= 1 - \Phi\left(\frac{-\mathbb{E}(\text{DV}|H_y)}{\sqrt{\text{Var(DV)}}}\right)\end{aligned}$$

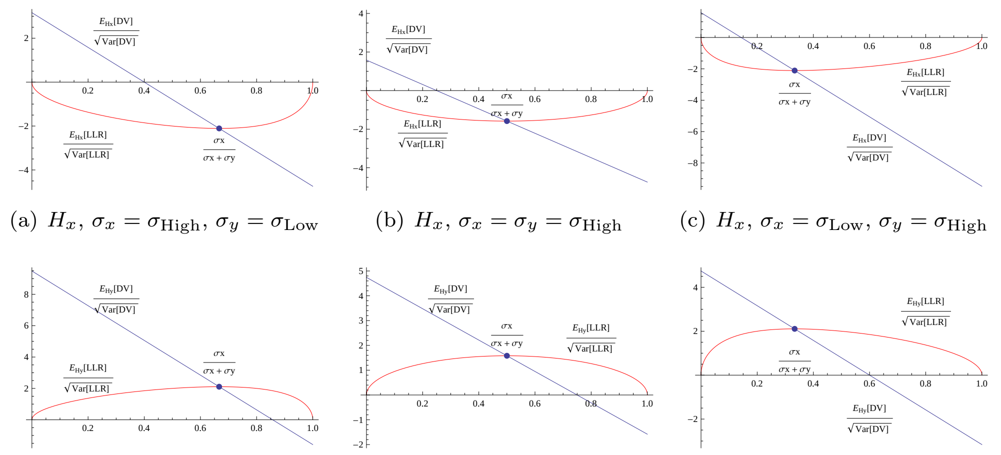

(a) H_x, $\sigma_x = \sigma_{\text{High}}$, $\sigma_y = \sigma_{\text{Low}}$ (b) H_x, $\sigma_x = \sigma_y = \sigma_{\text{High}}$ (c) H_x, $\sigma_x = \sigma_{\text{Low}}$, $\sigma_y = \sigma_{\text{High}}$

(d) H_y, $\sigma_x = \sigma_{\text{High}}$, $\sigma_y = \sigma_{\text{Low}}$ (e) H_y,$\sigma_x = \sigma_y = \sigma_{\text{High}}$ (f) H_y,$\sigma_x = \sigma_{\text{Low}}$, $\sigma_y = \sigma_{\text{High}}$

Figure 5.1: Plots comparing the argument of the DV (blue lines) and LLR (red lines) error functions across the interval of valid sampling strategies $[0, 1]$ under a number of parameterisations of the decision problem. In each plot the optimal sampling strategy $q = \frac{\sigma_x}{\sigma_x+\sigma_y}$ is marked on both the argument and derivative plots. Plots have been generated with $\mu_{\text{High}} = 3.0$, $\mu_{\text{Low}} = 1.0$, $\sigma_{\text{High}} = 2.0$, $\sigma_{\text{Low}} = 1.0$ and $T = 10$. From left to right the variances of the alternatives vary in each of the plots with Figures 1(a) and 1(d) having $\sigma_x = \sigma_{\text{High}}$ and $\sigma_y = \sigma_{\text{Low}}$, Figures 1(b) and 1(e) having $\sigma_x = \sigma_y = \sigma_{\text{High}}$ and in Figures 1(c) and 1(f) $\sigma_x = \sigma_{\text{Low}}$ and $\sigma_y = \sigma_{\text{High}}$. From top to bottom the means of the alternatives vary with Figures 1(a), 1(b) and 1(c) having $\mu_x = \mu_{\text{High}}$ and $\mu_y = \mu_{\text{Low}}$ and Figures 1(d), 1(e) and 1(f) having $\mu_x = \mu_{\text{Low}}$ and $\mu_y = \mu_{\text{High}}$. Comparing the DV and LLR plots it can be seen that, as expected, the two values are coincident at $q = \frac{\sigma_x}{\sigma_x+\sigma_y}$. Furthermore, from inspection of the DV argument plot (blue line), it can be seen that as the plot is a straight line, the derivative has a constant value across $[0, 1]$.

As $\Phi\left(.\right)$ is a monotonically increasing function, the error rate of the DV decision maker increases with the argument of $\Phi\left(.\right)$ under hypothesis H_x and decreases as the argument increases under hypothesis H_y. From Figure 5.1 it can be seen that for the examples six plotted the value of $\frac{-\mathbb{E}(\text{DV}|H_x)}{\sqrt{\text{Var}(\text{DV})}}$ decreases as the the q value is increased. Thus it appears the effect of deviating from the optimal sampling strategy $q^* = \frac{\sigma_x}{\sigma_x+\sigma_y}$ is to to bias the decision maker towards selecting the source from which more samples were drawn than stipulated by q^*.

To determine if this relationship holds for all decision problems, and not just the specific cases in Figure 5.1, further investigation is required. To this end we take the derivative

of $\frac{d}{dq}\frac{-\mathbb{E}(\mathrm{DV})}{\sqrt{\mathrm{Var}(\mathrm{DV})}}$ with respect to the sampling strategy q which yields the following result:

$$\frac{d}{dq}\frac{-\mathbb{E}(\mathrm{DV})}{\sqrt{\mathrm{Var}(\mathrm{DV})}} = \frac{(qT\mu_X - (1-q)Tw\mu_Y)(T\sigma_x^2 - Tw^2\sigma_y^2)}{2(qT\sigma_x^2 + w^2(1-q)T\sigma_y^2)^{\frac{3}{2}}} - \frac{(T\mu_X + Tw\mu_Y)}{\sqrt{(qT\sigma_x^2 + w^2(1-q)T\sigma_y^2)}} \tag{5.5}$$

Now setting $\frac{d}{dq}\frac{-\mathbb{E}(\mathrm{DV})}{\sqrt{\mathrm{Var}(\mathrm{DV})}} < 0$ we can begin to prove the relationship identified above.

$$0 > \frac{d}{dq}\frac{-\mathbb{E}(\mathrm{DV})}{\sqrt{\mathrm{Var}(\mathrm{DV})}}$$

$$\frac{(T\mu_X + Tw\mu_Y)}{\sqrt{(qT\sigma_x^2 + w^2(1-q)T\sigma_y^2)}} > \frac{(qT\mu_X - (1-q)Tw\mu_Y)(T\sigma_x^2 - Tw^2\sigma_y^2)}{2(qT\sigma_x^2 + w^2(1-q)T\sigma_y^2)^{\frac{3}{2}}}$$

Multiplying out the denominators and simplifying yields:

$$(qT\mu_X - (1-q)Tw\mu_Y)(T\sigma_x^2 - Tw^2\sigma_y^2) = 2(T\mu_X + Tw\mu_Y)(qT\sigma_x^2 + w^2(1-q)T\sigma_y^2)$$

$$(q\mu_X - (1-q)w\mu_Y)(\sigma_x^2 - w^2\sigma_y^2) = 2(\mu_X + w\mu_Y)(q\sigma_x^2 + w^2(1-q)\sigma_y^2)$$

Rearranging, the sampling strategy q can be written in terms of the remaining parameters:

$$\frac{w\mu_y\sigma_x^2 + 2w^2\mu_x\sigma_y^2 + w^3\mu_y\sigma_y^2}{(\mu_x + w\mu_y)} > q\left(w^2\sigma_y^2 - \sigma_x^2\right)$$

Simplifying further there are three distinct cases that must be handled depending on the value of $w^2\sigma_y^2 - \sigma_x^2$. First, when $w^2\sigma_y^2 = \sigma_x^2$ the RHS of the above reduces to 0 yielding:

$$\frac{w\mu_y\sigma_x^2 + 2w^2\mu_x\sigma_y^2 + w^3\mu_y\sigma_y^2}{(\mu_x + w\mu_y)} > 0$$

Next, when $w^2\sigma_y^2 > \sigma_x^2$ division by $w^2\sigma_y^2 - \sigma_x^2$ yields the following result:

$$\frac{w\mu_y\sigma_x^2 + 2w^2\mu_x\sigma_y^2 + w^3\mu_y\sigma_y^2}{(\mu_x + w\mu_y)\left(w^2\sigma_y^2 - \sigma_x^2\right)} > 1 > q$$

Finally, when $w^2\sigma_y^2 < \sigma_x^2$ division by $w^2\sigma_y^2 - \sigma_x^2$ yields the following result:

$$q > -1 > \frac{w\mu_y\sigma_x^2 + 2w^2\mu_x\sigma_y^2 + w^3\mu_y\sigma_y^2}{(\mu_x + w\mu_y)\left(w^2\sigma_y^2 - \sigma_x^2\right)}$$

With w, μ_x, μ_y, σ_x^2 and σ_y^2 all strictly positive, it can be seen that all three of these inequalities are necessarily satisfied. Since all of these cases completely encompass the interval $q \in [0,1]$, all valid sampling strategies yield a negative derivative, regardless of hypothesis. Thus the effect of deviating from the optimal sampling strategy $q^* = \frac{\sigma_x}{\sigma_x + \sigma_y}$ is to bias the decision maker towards selecting the source from which more samples were drawn than stipulated by q^*.

References

[1] BJ Flehinger and TA Louis. Sequential medical trials with data dependent treatment allocation. *Proc. 6th Berkeley Symp*, pages 43–51, 1972.

[2] BJ Flehinger, TA Louis, Herbert Robbins, and BH Singer. Reducing the number of inferior treatments in clinical trials. *Proceedings of the National Academy of Sciences*, 69(10):2993, 1972.

[3] L. S. Hayre. Two-Population Sequential Tests with Three Hypotheses. *Biometrika*, 66(3):465, December 1979.

[4] Lakhbir S. Hayre and John C. Gittins. Sequential Selection of the Larger of Two Normal Means. *Journal of the American Statistical Association*, 76(375):696, September 1981.

[5] LS Hayre. Sequential estimation of the difference between the means of two normal populations. *Metrika*, 30(6):101–107, 1983.

[6] SH Kim and AS Cohen. On the Behrens-Fisher problem: A review. *Journal of Educational and Behavioral ...*, 1998.

[7] Mary Natrella. *NIST/SEMATECH e-Handbook of Statistical Methods.* NIST/SEMATECH, July 2010.

[8] J. Neyman and E. S. Pearson. On the problem of the most efficient tests of statistical hypotheses. *Philosophical Transactions of the Royal Society of London. Series A, Containing Papers of a Mathematical or Physical Character*, 231(1933):289–337, 1933.

[9] William H. Press, Brian P. Flannery, Saul A. Teukolsky, and William T. Vetterling. *Numerical Recipes in Fortran: The Art of Scientific Computing*, chapter 2.3 LU Decomposition and Its Applications. Cambridge University Press, 2 edition, September 1992.

[10] Robert H Wurtz. Neuronal mechanisms of visual stability. *Vision research*, 48(20):2070–89, September 2008.
